# Supplementary material for: Combined inhibition of focal adhesion kinase and RAF/MEK elicits synergistic inhibition of melanoma growth and reduces metastases
Source: Cell Rep Med. 2025 Feb 7;6(2):101943. doi: 10.1016/j.xcrm.2025.101943 (PMC11866499; doi:10.1016/j.xcrm.2025.101943)
Supplement: Document S1. Figures S1–S4 [file mmc1.pdf]

Cell Reports Medicine, Volume *6*

## Supplemental information

# Combined inhibition of focal adhesion kinase and RAF/MEK elicits synergistic inhibition of melanoma growth and reduces metastases

**Jared Almazan, Tursun Turapov, David A. Kircher, Karly A. Stanley, Katie Culver, A. Paulina Medellin, MiKaela N. Field, Gennie L. Parkman, Howard Colman, Silvia Coma, Jonathan A. Pachter, and Sheri L. Holmen**

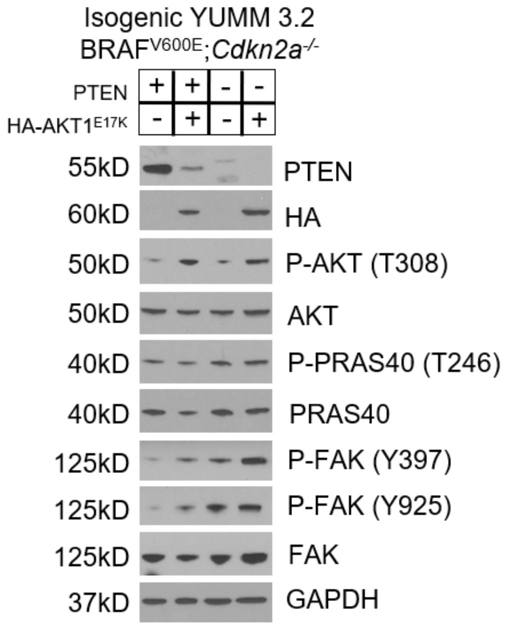


**Supplemental Figure 1 title:**

YUMM3.2 cells that express AKT$^{E17K}$ and/or are deficient in PTEN show hyperactivity of the PI3K/AKT pathway *in vitro*, Related to Figure 1.

**Supplemental Figure 1 legend:**

Immunoblotting was used to quantify levels of PTEN, HA (AKT$^{E17K}$), P-AKT (T308), AKT, P-PRAS40 (T246), PRAS40, P-FAK (Y397, Y925), FAK, and GAPDH (loading control) in four isogenic YUMM3.2 cell lines +/- AKT1$^{E17K}$ and +/- PTEN.

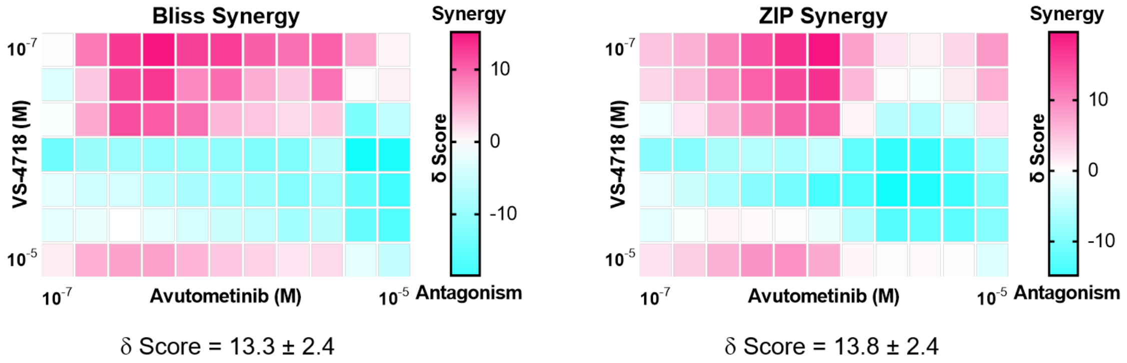


**Supplemental Figure 2 title:**

Combined FAK/RAF/MEK inhibition reduces YUMM3.2;Pten-/-;AKT1$^{E17K}$ cell proliferation *in vitro*, Related to Figure 1.

**Supplemental Figure 2 legend:**

Cell viability was assessed in YUMM3.2;Pten-/-;AKT1$^{E17K}$ cells treated with increasing concentrations of VS-4718 and avutometinib for 72 hours. Drug synergy was measured using Bliss and Zero Interaction Potency (Zip) tests with increasing concentrations of VS-4718 and avutometinib in combination; values greater than zero denote synergy.

**A.**

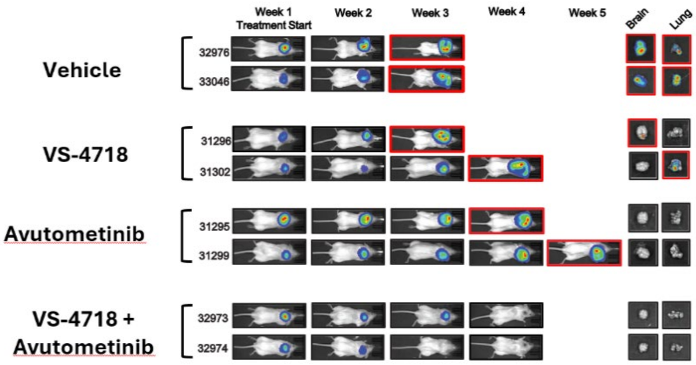


**B.**

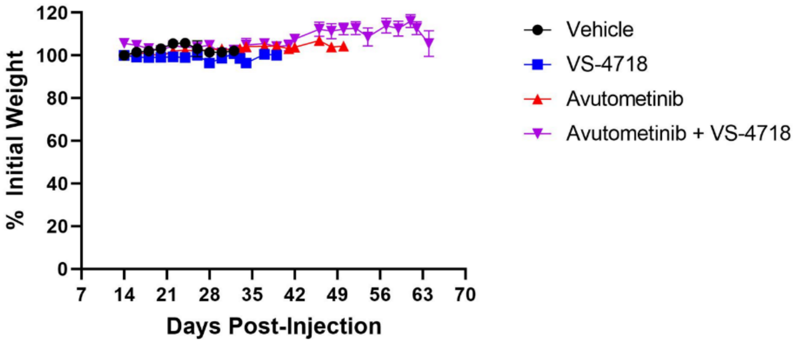


**Supplemental Figure 3 title:**

Combined FAK/RAF/MEK inhibition diminishes or eliminates YUMM3.2;Pten-/-;AKT1$^{E17K}$ bioluminescence signal in the lungs and brains of mice, Related to Figure 4.

**Supplemental Figure 3 legend:**

**A,** Bioluminescence imaging (BLI) of representative 7-9 week old C57BL/6 glowing head mice subcutaneously injected with luciferase-expressing YUMM3.2;Pten-/-;AKT1$^{E17K}$ cells. Mice were treated with vehicle, VS-4718, avutometinib, or VS-4718 + avutometinib for 28 days. BLI on the lungs and brains of mice was performed *ex vivo*, following the injection of luciferin. Red outline denotes endpoint BLI. **B,** Mean weight of mice +/-SEM by cohort over time, post-injection; vehicle (black) n=8, VS-4718 (blue) n=13, avutometinib (red) n=9, VS-4718 + avutometinib (purple) n=6.

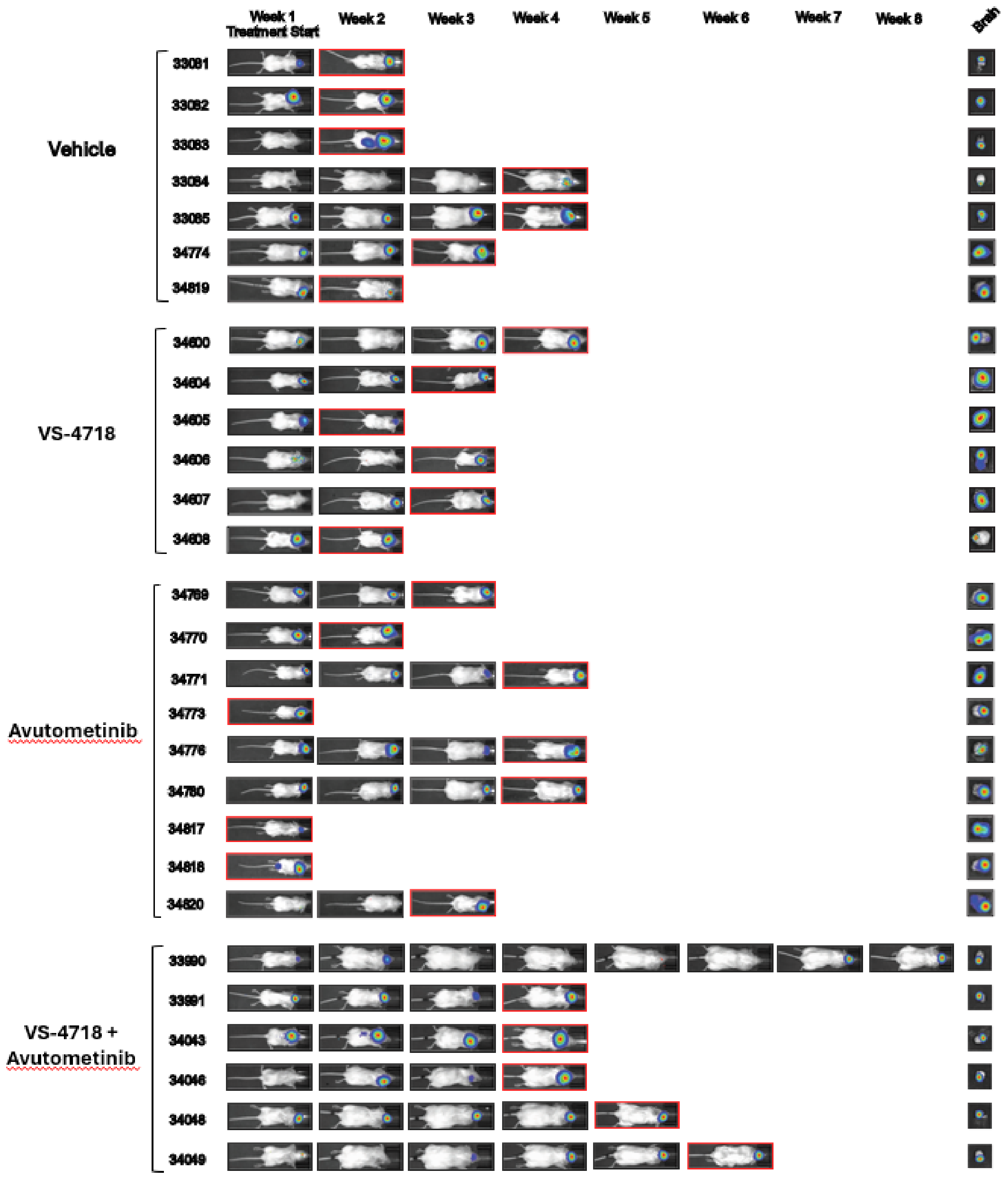


**Supplemental Figure 4 title:**

Combined FAK/RAF/MEK inhibition diminishes YUMM3.2;Pten-/-;AKT1$^{E17K}$ bioluminescence signal in existing brain metastases, Related to Figure 5.

**Supplemental Figure 4 legend:**

Bioluminescence imaging (BLI) of newborn C57BL/6 glowing head mice intracranially injected with luciferase-expressing YUMM3.2;Pten-/-;AKT1$^{E17K}$ cells. Mice were treated with vehicle, VS-4718, avutometinib, or VS-4718 + avutometinib for 28 days upon weaning. BLI on the brains of mice was performed *ex vivo*, following the injection of luciferin. Red outline denotes endpoint BLI.
