# Supplementary material for: Combined inhibition of focal adhesion kinase and RAF/MEK elicits synergistic inhibition of melanoma growth and reduces metastases
Source: Cell Rep Med. 2025 Feb 7;6(2):101943. doi: 10.1016/j.xcrm.2025.101943 (PMC11866499; doi:10.1016/j.xcrm.2025.101943)
Supplement: Document S2. Article plus supplemental information [file mmc2.pdf]

Cell Reports
Medicine

Article

Combined inhibition of focal adhesion kinase and RAF/MEK elicits synergistic inhibition of melanoma growth and reduces metastases

Graphical abstract

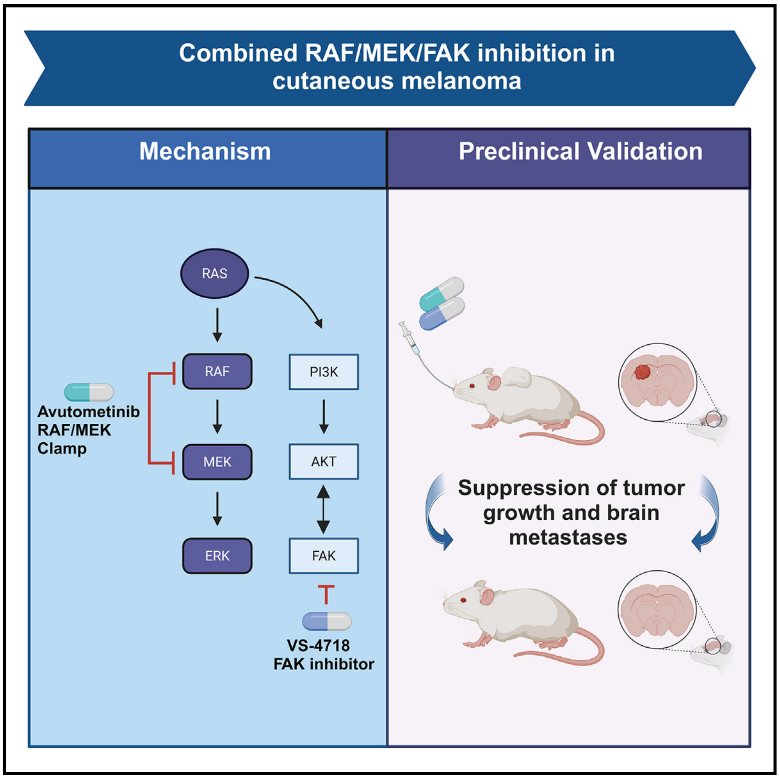

Highlights

- FAK/RAF/MEK inhibition synergistically induces apoptosis in melanoma cells *in vitro*
- FAK inhibition directly reduces brain metastasis development *in vivo*
- The combination is well tolerated and promotes tumor regression *in vivo*
- FAK/RAF/MEK inhibition prolongs survival in mice with existing brain metastases

Authors

Jared Almazan, Tursun Turapov, David A. Kircher, ..., Silvia Coma, Jonathan A. Pachter, Sheri L. Holmen

Correspondence

sheri.holmen@hci.utah.edu

In brief

Patients with melanoma brain metastases have a poor prognosis and limited treatment options. Here, Almazan et al. demonstrate the synergistic effects of targeting focal adhesion kinase (FAK) and RAF/MEK to inhibit melanoma growth and metastasis. These findings highlight the therapeutic potential of targeting both FAK and RAF/MEK signaling in this disease.

Almazan et al., 2025, Cell Reports Medicine *6*, 101943
February 18, 2025 © 2025 The Authors. Published by Elsevier Inc.
https://doi.org/10.1016/j.xcrm.2025.101943

CellPress

Cell Reports Medicine

CellPress
OPEN ACCESS

Article

Combined inhibition of focal adhesion kinase and RAF/MEK elicits synergistic inhibition of melanoma growth and reduces metastases

Jared Almazan,[1,2] Tursun Turapov,[1,3] David A. Kircher,[1,3] Karly A. Stanley,[1,2] Katie Culver,[1,3] A. Paulina Medellin,[1,3] MiKaela N. Field,[1,3] Gennie L. Parkman,[1,4] Howard Colman,[1,5] Silvia Coma,[6] Jonathan A. Pachter,[6] and Sheri L. Holmen[1,2,3,7,*]
[1]Huntsman Cancer Institute, University of Utah Health Sciences Center, Salt Lake City, UT 84112, USA
[2]Department of Oncological Sciences, University of Utah Health Sciences Center, Salt Lake City, UT 84112, USA
[3]Department of Surgery, University of Utah Health Sciences Center, Salt Lake City, UT 84112, USA
[4]Department of Zoology, Weber State University, Ogden, UT 84408, USA
[5]Department of Neurosurgery, University of Utah Health Sciences Center, Salt Lake City, UT 84112, USA
[6]Verastem Oncology, Needham, MA 02494, USA
[7]Lead contact
*Correspondence: sheri.holmen@hci.utah.edu
https://doi.org/10.1016/j.xcrm.2025.101943

SUMMARY

This study addresses the urgent need for effective therapies for patients with brain metastases from cutaneous melanoma, a major cause of treatment failure despite recent therapeutic advances. Utilizing mouse models that mimic human melanoma brain metastases, this study investigates the necessity of focal adhesion kinase (FAK) in the development of distant metastases and its potential as a therapeutic target. Pharmacological inhibition of FAK demonstrates significant efficacy in reducing the development of brain metastases in preclinical mouse models. Importantly, the study provides insight into the crosstalk between FAK and mitogen-activated protein kinase (MAPK) pathway signaling and highlights the synergistic effects of combined inhibition of FAK, rapidly accelerated fibrosarcoma (RAF), and mitogen-activated protein kinase kinase (MEK) in cutaneous melanoma. These findings provide the rationale for clinical evaluation of the efficacy of the FAK inhibitor defactinib and the RAF/MEK inhibitor avutometinib in patients with brain metastases from cutaneous melanoma.

INTRODUCTION

Since 2011, several therapies have been Food and Drug Administration (FDA) approved for melanoma, but brain metastases are often the major cause of treatment failure. Melanoma patients with brain metastases have a dismal prognosis and median overall survival is only about 1 year from the time of diagnosis.[1] Given this grim prognosis, effective therapeutic approaches are urgently needed for these patients. A major challenge in developing effective treatments for melanoma brain metastases has been the lack of relevant preclinical models that mimic metastatic patterns observed in patients. Using data obtained from human melanoma samples, which demonstrated increased levels of phosphorylated AKT (P-AKT) and decreased levels of phosphatase and TENsin homolog deleted on chromosome 10 (PTEN) in brain metastases,[2–4] we generated a mouse model of melanoma with hyperactivation of AKT1 signaling that develops lung and brain metastases similar to the human disease. We used this model to delineate the mechanisms by which AKT promotes metastasis and evaluated whether this could be exploited therapeutically.[5]

Historically, the use of pharmacological AKT inhibitors in melanoma clinical trials has had limited efficacy.[6,7] Interestingly, we observed that pharmacological inhibition of AKT had no effect on BRAF-mutant melanoma cell proliferation *in vitro* or *in vivo* but genetic silencing of all three AKT paralogs produced profound melanoma cell death. This difference is due in part to relief of negative feedback signaling following AKT inhibition that paradoxically activates the pathway; this overwhelms pharmacological blockade but is halted when AKT is genetically silenced.[8] To bypass this effect and identify AKT1-specific effectors associated with the development of brain metastases, we used a proteomics approach to assess differences between non-metastatic and brain metastatic melanomas. We observed that BRAF-mutant melanoma cells expressing an active AKT1 mutant (E17K) displayed elevated levels of focal adhesion (FA) factors and phosphorylated focal adhesion kinase (P-FAK). $AKT1^{E17K}$ expression in melanoma cells increased invasion, and this was reduced by pharmacological inhibition of either AKT or focal adhesion kinase (FAK) *in vitro*.[5] FAK is a non-receptor tyrosine kinase that promotes cancer cell motility and has also been implicated in promoting cell proliferation, invasion, and metastasis (reviewed by Chuang et al.[9]).

Notably, recent studies have underscored the role of FAK in mediating resistance to targeted therapies, particularly those

This is an open access article under the CC BY license (http://creativecommons.org/licenses/by/4.0/).

that inhibit the mitogen-activated protein kinase (MAPK) pathway.[10–15] Inhibition of FAK in combination with RAF/MEK inhibition has been shown to effectively overcome this resistance, resulting in improved anti-tumor responses in preclinical models. A study by Chen et al. demonstrated significant tumor growth inhibition in BRAF inhibitor (BRAFi)-resistant colorectal cancer when FAK inhibitors (FAKi) were combined with RAF/MEK inhibitors (RAFi/MEKi). The preclinical models used included cell line xenografts and patient-derived xenografts.[10] Shapiro et al. showed similar results in malignant pleural mesothelioma, particularly in the context of Merlin deficiency, using xenograft models in mice.[14] Yoshimura et al. and McNamara et al. found that combining FAKi with the RAFi/MEKi avutometinib significantly reduced tumor growth in Kirsten rat sarcoma viral oncogene homolog (KRAS)-mutated non-small cell lung cancer (NSCLC) and low-grade serous ovarian cancer (LGSOC), respectively, using cell line-derived xenografts and patient-derived xenografts in mice.[12,15] Hartwich et al. further supported these findings by demonstrating that the combination of avutometinib and the FAKi defactinib led to significant tumor growth inhibition in high-grade endometrioid endometrial cancer using cell line and patient-derived xenografts.[16] Similarly, Demirkiran et al. showed that this combination was effective in uterine carcinosarcomas, using both *in vitro* and *in vivo* models.[17] These studies highlight the potential of this combination therapy to enhance treatment efficacy across a variety of malignancies.

FAK-induced resistance to MAPK inhibition also extends to cutaneous melanoma (CM), where Hirata et al. and Pang et al. found that intrinsic activation of FAK can mediate resistance to the BRAFi PLX4720 and vemurafenib, respectively, by reactivating MAPK pathway signaling. Their studies demonstrated that combining BRAF and FAK inhibition in patient-derived CM xenografts led to prolonged tumor control, even after resistance to BRAFi developed.[11,13] This suggests that FAK plays a crucial role in adaptive resistance mechanisms in CM, positioning it as a compelling target for combination therapies for this indication.

There are several FAKi in clinical trials with the most advanced compound being defactinib (Verastem Oncology), which has been assessed as both monotherapy and in combination with other drugs in patients with solid tumors (reviewed by Dawson et al.[18]). As a monotherapy, defactinib demonstrated clinical activity in heavily pre-treated KRAS-mutant NSCLC.[19] Defactinib also showed a tolerable safety profile both as a single agent and in combination with gemcitabine and pembrolizumab or avutometinib.[19–21] Avutometinib is a RAF/MEK clamp that induces formation of inactive complexes of MEK with ARAF, BRAF, and CRAF, which leads to more complete and durable anti-tumor responses through maximal MAPK pathway inhibition. In contrast to other MEKi, avutometinib blocks both MEK kinase activity and the ability of RAF to phosphorylate MEK. This mechanism allows avutometinib to block MEK signaling without the compensatory activation of MEK that appears to limit the efficacy of other inhibitors.[22,23]

In 2021, the FDA granted Breakthrough Therapy designation for the combination of defactinib with avutometinib for the treatment of patients with recurrent LGSOC after one or more prior lines of systemic therapy. This combination is currently being evaluated in recurrent LGSOC in a phase 2 study (RAMP 201; NCT04625270) and in a phase 3 study (RAMP 301; NCT06072781). Interim data from RAMP 201 demonstrated a confirmed overall response rate of 45% (13/29; 95% confidence interval [CI]: 26%, 64%), and tumor shrinkage was observed in the vast majority of LGSOC patients (86%; 25/29). The majority of adverse events were grade 1–2, and a limited number of patients experienced dose reductions or discontinuations for adverse events.[20,24]

In this study, we investigated the necessity of FAK in the development of distant metastases and explored the therapeutic efficacy of FAK inhibition, alone and in combination with RAF/MEK blockade. Our findings reveal that pharmacological inhibition of FAK in combination with RAF/MEK inhibition significantly suppresses melanoma growth, reduces the development of metastases in preclinical mouse models, and prolongs survival in mice with existing brain metastases. Importantly, we provide insight into the crosstalk between the FAK and MAPK signaling pathways and demonstrate the synergistic effects of combined inhibition. These results underscore the therapeutic potential of targeting the FAK signaling axis in combination with blockade of MAPK signaling in cutaneous melanoma.

RESULTS

Combined inhibition of RAF, MEK, and FAK abrogates melanoma cell growth *in vitro*

To determine the effect of FAK inhibition alone and in combination with MAPK pathway blockade *in vitro*, we utilized YUMM3.2 cells that express BRAF^{V600E} and are deficient for *Cdkn2a*.[25] These cells were derived from a female mouse and are syngeneic with the C57BL/6 mouse strain. We previously utilized CRISPR-CAS9 gene editing technology to generate a *Pten*-deficient isogenic variant of YUMM3.2.[8] These YUMM3.2 isogenic cells were further modified by infection with a lentivirus harboring Akt1^{E17K} and co-expressing luciferase and EGFP (YUMM3.2;Pten$^{-/-}$;Akt1^{E17K}). Loss of Pten and gain of Akt1^{E17K} expression was confirmed by immunoblotting (Figure S1). In agreement with our prior findings in YUMM1.1 cells, which also express BRAF^{V600E} and are deficient for *Cdkn2a* and *Pten*,[5] expression of Akt1^{E17K} led to increased P-AKT at Threonine 308 and cooperated with loss of Pten to increase P-FAK at tyrosine residues 397 and 925 (Figure S1).

To determine whether FAK and/or RAF/MEK inhibition reduces melanoma cell proliferation, YUMM3.2;Pten$^{-/-}$;Akt1^{E17K} cells were treated with increasing doses of either VS-4718, which is an ATP-competitive FAKi and surrogate for defactinib, or avutometinib for 72 h. Cell confluence was assessed every 2 h using the Incucyte S3 live-cell analysis instrument. VS-4718 reduced proliferation at concentrations equal to and above 2.5 μM (Figure 1A), and avutometinib decreased proliferation at concentrations equal to and higher than 625 nM (Figure 1B). To define the half-maximal inhibitory concentration (IC50) of each drug alone and in combination, these cells were treated in triplicate with increasing concentrations of VS-4718, avutometinib, or the combination of VS-4718 and avutometinib. After 72 h, cell viability was assessed using the ATPlite cell viability assay. The IC50 of VS-4718 was determined to be 1.34 μM, the IC50 of avutometinib was 256 nM, and the IC50 of the combination was 142 nM (Figure 1C).

CellPress
OPEN ACCESS

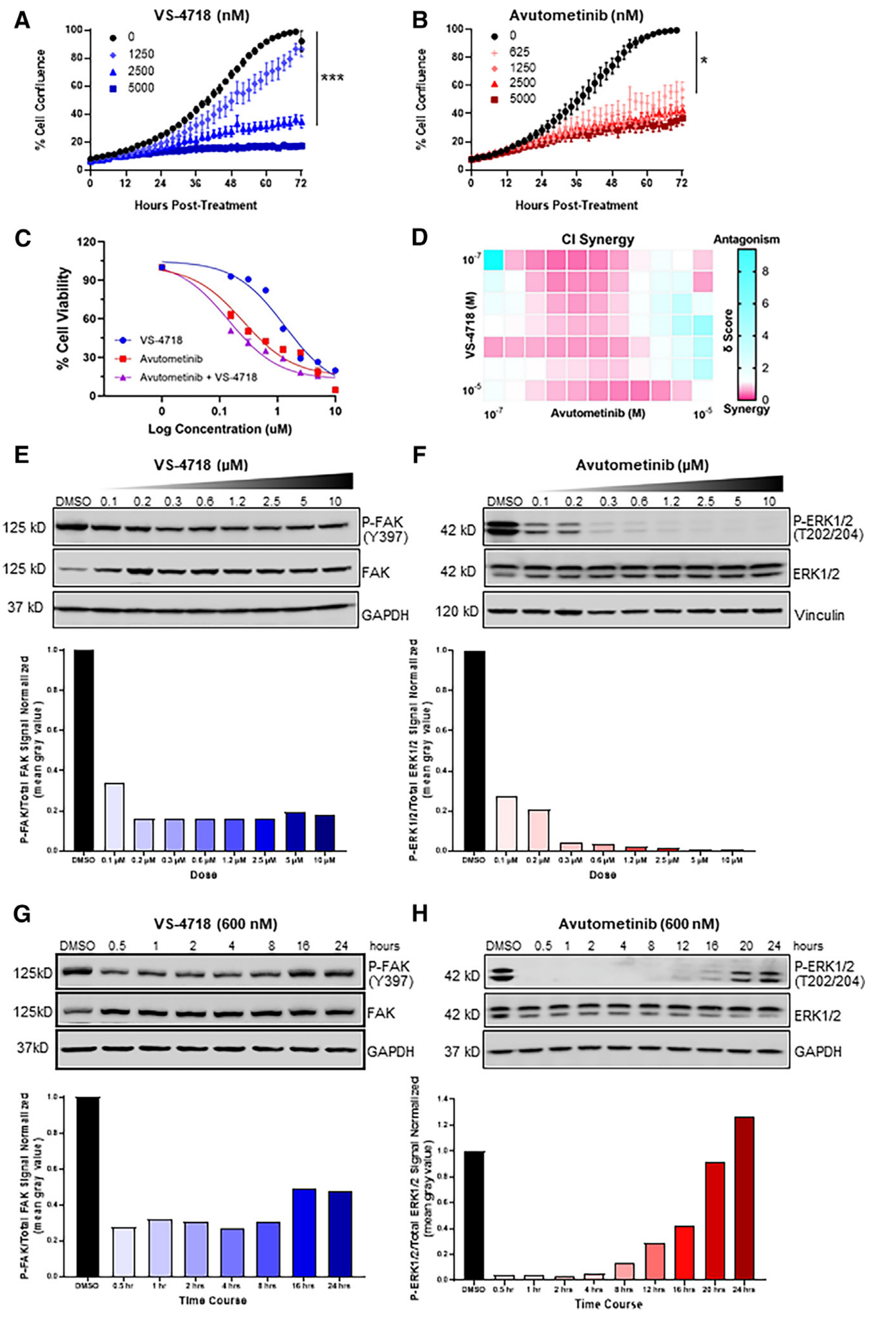

Figure 1. Combined inhibition of RAF, MEK, and FAK abrogates melanoma cell growth *in vitro*

(A and B) Cell proliferation was measured in YUMM3.2;Pten$^{-/-}$;Akt1^{E17K} cells treated in triplicate with increasing concentrations of VS-4718 (blue) or avutometinib (red). A Dunnett's multiple comparison test was used to determine statistical significance (72 h). Mean values +/− SEM are reported. p values are as follows: $p < 0.05$ (*), $p < 0.001$ (***).

(C) IC50 values of VS-4718- and avutometinib-treated cells.

(D) Drug synergy was measured in duplicate using the Chou-Talalay combination index (CI) for increasing concentrations of VS-4718 and avutometinib in combination; values less than 1 denote synergy.

(E and F) Immunoblotting was used to quantify levels of total FAK and P-FAK (Y397) in cells treated with increasing concentrations of VS-4718 and total ERK and P-ERK1/2 (T202/204) in cells treated with increasing concentrations of avutometinib for 2 h. P-FAK was normalized to total FAK, and P-ERK1/2 was normalized to total ERK. Histogram values are expressed as fold change relative to DMSO control.

(G and H) Immunoblotting was used to quantify levels of total FAK and P-FAK (Y397) in cells treated with 600 nM of VS-4718 and total ERK and P-ERK1/2 (T202/204) in cells treated with 600 nM of avutometinib over 24 h. P-FAK was normalized to total FAK, and P-ERK1/2 was normalized to total ERK. Histogram values are expressed as fold change relative to DMSO control.

See also Figure S1 and S2.

To determine if FAK inhibition synergistically cooperates with RAF/MEK inhibition to reduce cell proliferation, YUMM3.2; Pten$^{-/-}$;Akt1^{E17K} cells were treated with different concentrations of VS-4718 and avutometinib. The cells were assayed using the ATPlite cell viability assay to assess drug synergy or antagonism using multiple methods including the Chou-Talalay combination index (CI) where CI < 1, = 1, and > 1 indicates synergism, additive effect, or antagonism, respectively (reviewed by Duarte et al.[26]). Bliss and zero interaction potency (Zip) methods were also used where values > 0 indicate synergism. The drug combination was synergistic at several dose levels as assessed by CI, Bliss, and Zip (Figure 1D and Figure S2).

To assess target inhibition following drug treatment, YUMM3.2;Pten$^{-/-}$; Akt1^{E17K} cells were treated with increasing concentrations of VS-4718. After 2 h, lysates were collected and immunoblotted for P-FAK (Y397), total FAK, and glyceraldehyde 3-phosphate dehydrogenase (GAPDH) as a loading control. Band intensity was quantified using ImageJ, and P-FAK was normalized to total FAK. When compared with the DMSO control, P-FAK was reduced by >80% at concentrations of 0.2 μM and higher (Figure 1E). YUMM3.2;Pten$^{-/-}$;Akt1^{E17K} cells were also treated with increasing concentrations of avutometinib. After 2 h, lysates were collected and immunoblotted for phosphorylated ERK1/2 (P-ERK1/2; T202/204), total ERK, and vinculin as a loading control. When compared with the DMSO control, P-ERK1/2 was reduced by > 95% at concentrations of 0.3 μM and higher (Figure 1F). We next performed a 24 h time course experiment evaluating VS-4718 and avutometinib target inhibition at a dose of 600 nM. Maximum inhibition of P-FAK by

VS-4718 occurred between 30 min and 4 h. By 16 h, the levels of P-FAK began to recover (Figure 1G). Maximum inhibition of P-ERK1/2 by avutometinib occurred between 30 min and 12 h. By 16 h, the levels of P-ERK1/2 began to recover (Figure 1H).

Combined inhibition of RAF, MEK, and FAK increases melanoma cell death *in vitro*

Informed by the dose response and synergy experiments, YUMM3.2;Pten$^{-/-}$;Akt1^{E17K} cells were treated with 600 nM of avutometinib and/or VS-4718 and cell viability was assessed after 72 h. The FDA-approved mutant BRAFi encorafenib was included for comparison. The combination of VS-4718 and avutometinib decreased cell viability more effectively than either VS-4718 alone ($p \leq 0.0001$) or avutometinib alone ($p \leq 0.001$) and was as effective as the combination of VS-4718 and encorafenib. The addition of encorafenib to the combination of avutometinib and VS-4718 did not enhance the efficacy of the VS-4718 and avutometinib combination *in vitro*. Target inhibition was confirmed for all drugs by immunoblotting for P-FAK (Y397 and Y925) and P-ERK1/2 (T202/204) (Figure 2). In agreement with findings from others (reviewed by Dawson et al.[18]), we observed increased P-FAK when the MAPK pathway was inhibited with avutometinib or encorafenib. In addition, we observed decreased P-AKT (T308 and S473) when FAK was inhibited. Cleaved caspase-3 was assessed as an indicator of apoptosis, and we observed that increased levels of cleaved caspase-3, detected by immunoblot, correlated with decreased cell viability (Figure 2).

Pharmacological inhibition of FAK reduces the development of brain metastases from primary melanoma tumors

To determine whether inhibition of FAK reduces the development of distant metastases, we utilized our autochthonous replication-competent Avian sarcoma leukosis virus (ASLV) long-terminal repeat (LTR) with a splice acceptor (RCAS)/tumor virus A (TVA) avian retroviral melanoma model driven by BRAF^{V600E} and myristoylated (myr) AKT1, in the context of *Pten* and *Cdkn2a* loss as previously described[27] (Figure 3A). Two different ATP-competitive FAKi, VS-4718 and PF-573228, were compared against a vehicle control cohort and a standard-of-care cohort for BRAF-mutant CM, consisting of the BRAFi encorafenib in combination with the MEKi binimetinib. Melanomas were induced in newborn *Dct::TVA;Braf*^{CA}*;Cdkn2a*$^{lox/lox}$*;Pten*$^{lox/lox}$ mice through injection of avian DF-1 fibroblasts harboring RCAS-Cre and RCAS-myrAKT1. At weaning, mice were randomized into four cohorts: vehicle, PF-573228, VS-4718, or encorafenib in combination with binimetinib. Mice were treated for 28 days and monitored for primary tumor formation, and measurements were taken three times weekly once tumors were palpable. Administration of encorafenib and binimetinib predictably delayed primary tumor onset and growth, whereas tumors developed unimpeded in the presence of either FAKi (Figures 3B and 3C). Mice were sacrificed when tumors reached ~10% body weight or when mice appeared distressed. Brains were collected and analyzed histologically to assess differences in metastasis rates between the cohorts (Figure 3D). Inhibition of either RAF/MEK or FAK significantly reduced the incidence of brain metastases compared with vehicle-treated mice ($p < 0.01$; Figure 3E). The reduction in brain metastasis by PF-573228 and VS-4718 suggests that FAKi have a specific and direct impact on the metastatic processes, even though they do not affect tumor onset or primary tumor growth.

Combined FAK/RAF/MEK inhibition significantly reduces tumor growth, prolongs overall survival, and reduces metastases in mice with established primary melanomas

To evaluate the efficacy of FAK inhibition alone or in combination with RAF/MEK inhibition in established tumors and in distant metastases that originated from these primary tumors, we utilized the YUMM3.2;Pten$^{-/-}$;Akt1^{E17K} cells, which express GFP and luciferase and are syngeneic with C57BL/6 mice. These cells were transplanted subcutaneously into 7- to 9-week-old C57BL/6 glowing head mice, which are tolerized to GFP and luciferase.[28] Once tumors were measurable, the mice were treated with vehicle, VS-4718, avutometinib, or the combination of VS-4718 and avutometinib for 28 days. Mice were imaged using the *in vivo* imaging system (IVIS) for bioluminescence imaging (BLI) to monitor luminescence, and tumors were measured with calipers three times weekly (Figure 4A). Mice were sacrificed when tumors reached ~10% body weight or when they appeared distressed. Although single agent treatment with VS-4718 or avutometinib significantly slowed tumor growth compared with vehicle-treated mice, tumors continued to progress throughout the treatment duration. In contrast, combined inhibition of FAK and RAF/MEK resulted in tumor regression in all treated mice (Figures 4B and 4C). Combined treatment with VS-4718 and avutometinib also significantly prolonged survival compared with either vehicle- or single-agent-treated mice (Figure 4D). Longitudinal and endpoint BLI were assessed for all mice and are shown for representative mice in Figure S3A. No treatment-related toxicity, as assessed by differences in body weight, were observed during the study period (Figure S3B). *Ex vivo* BLI was quantified for lungs and brains for all mice, and metastases were further assessed histologically. VS-4718 or avutometinib alone and in combination significantly reduced lung and brain metastases (Figure 4E). Immunoblotting was used to assess FAK and MAPK pathway inhibition in tumors at the end of the study. P-FAK was significantly reduced in both the VS-4718 single agent and combination cohorts. P-MEK and P-ERK were significantly reduced in both the single agent avutometinib samples and in the VS-4718 and avutometinib combination-treated samples (Figures 4F and 4G). Altogether, these data suggest that the combination of FAK and RAF/MEK inhibition significantly reduces primary tumor growth, prolongs overall survival, and reduces distant metastases in mice with existing primary melanoma tumors.

Combined FAK/RAF/MEK inhibition significantly prolongs survival in mice with existing brain metastases

Although treatment with VS-4718 and avutometinib in combination reduced lung and brain metastases in mice with established tumors, it was unclear whether the decrease in metastases in this cohort was due solely to the effects of the treatment on primary tumor growth. Therefore, to directly evaluate the effect of the drug treatment on existing melanoma cells in the brain, we developed a melanoma brain tumor model whereby YUMM3.2;Pten$^{-/-}$;Akt1^{E17K} cells were intracranially injected

CellPress
OPEN ACCESS

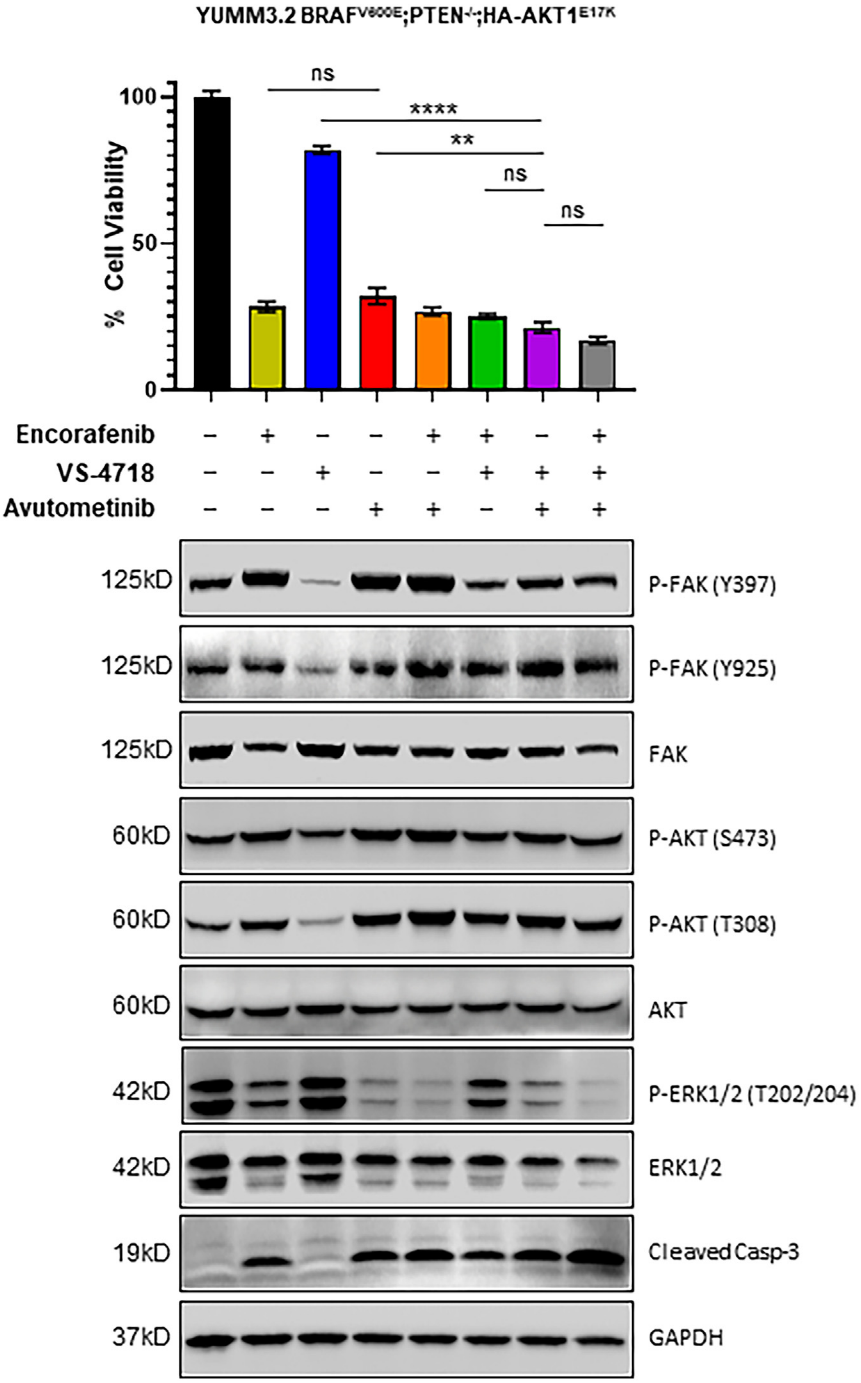

Figure 2. Combined inhibition of RAF, MEK, and FAK increases melanoma cell death *in vitro*

Cell viability was measured in YUMM3.2;Pten$^{-/-}$; AKT1^{E17K} cells treated in replicates of eight with 600 nM of encorafenib, avutometinib, and VS-4718 or a combination of each drug after 72 h. A one-way ANOVA was used to determine statistical significance. Mean values +/− SD are reported. p values are as follows: $p < 0.01$ (**), $p < 0.0001$ (****); ns, not significant. Immunoblotting was used to quantify levels of P-FAK (Y397, Y925), total FAK, P-AKT (S473, T308), total AKT, P-ERK1/2 (T202/204), total ERK1/2, cleaved caspase-3, and GAPDH (loading control).

into newborn glowing head C57BL/6 mice[28] (Figure 5A). All mice were imaged with the IVIS *in vivo* BLI system upon weaning and were subjected to a 28-day treatment of VS-4718 and avutometinib alone or in combination once a signal was detected. Mice were imaged weekly to monitor luminescence, and *ex vivo* brain BLI was performed upon euthanasia (Figure S4). Consistently, all mice in the vehicle, VS-4718, and avutometinib cohorts were sacrificed prior to the end of the treatment period due to declining health. In contrast, a third of the mice in the VS-4718 and avutometinib combination cohort survived past 28 days, amounting to a statistically significant increase in survival compared with the vehicle and monotherapy cohorts (Figure 5B).

The brains of all mice were collected and examined histologically following hematoxylin and eosin (H&E) staining (Figure 5C). Further analysis of brain tumors by immunohistochemistry (IHC) revealed the presence of P-FAK in vehicle or avutometinib cohorts and reduced P-FAK in those treated with VS-4718 alone or in combination with avutometinib. We also observed the presence of P-ERK in vehicle or VS-4718 cohorts and the absence of detectable P-ERK in those treated with avutometinib or VS-4718 in combination with avutometinib. Expression of mutant AKT was detected by IHC for the hemagglutinin (HA) epitope tag. Tumors were also stained for the proliferation marker Ki67, revealing a slight decrease in those treated with VS-4718 compared with the vehicle cohort and a pronounced decrease for those treated with avutometinib alone or in combination with VS-4718 (Figure 5C). Quantification of the P-ERK and P-FAK IHC stains is shown in Figure 5D. Altogether, this demonstrates that the combination of FAK and RAF/MEK inhibition prolongs overall survival in mice with existing brain metastases.

DISCUSSION

The emergence of both immunotherapy and targeted therapies has significantly improved outcomes for melanoma patients; however, the management of brain metastases remains a significant challenge due to their poor prognosis and limited treatment

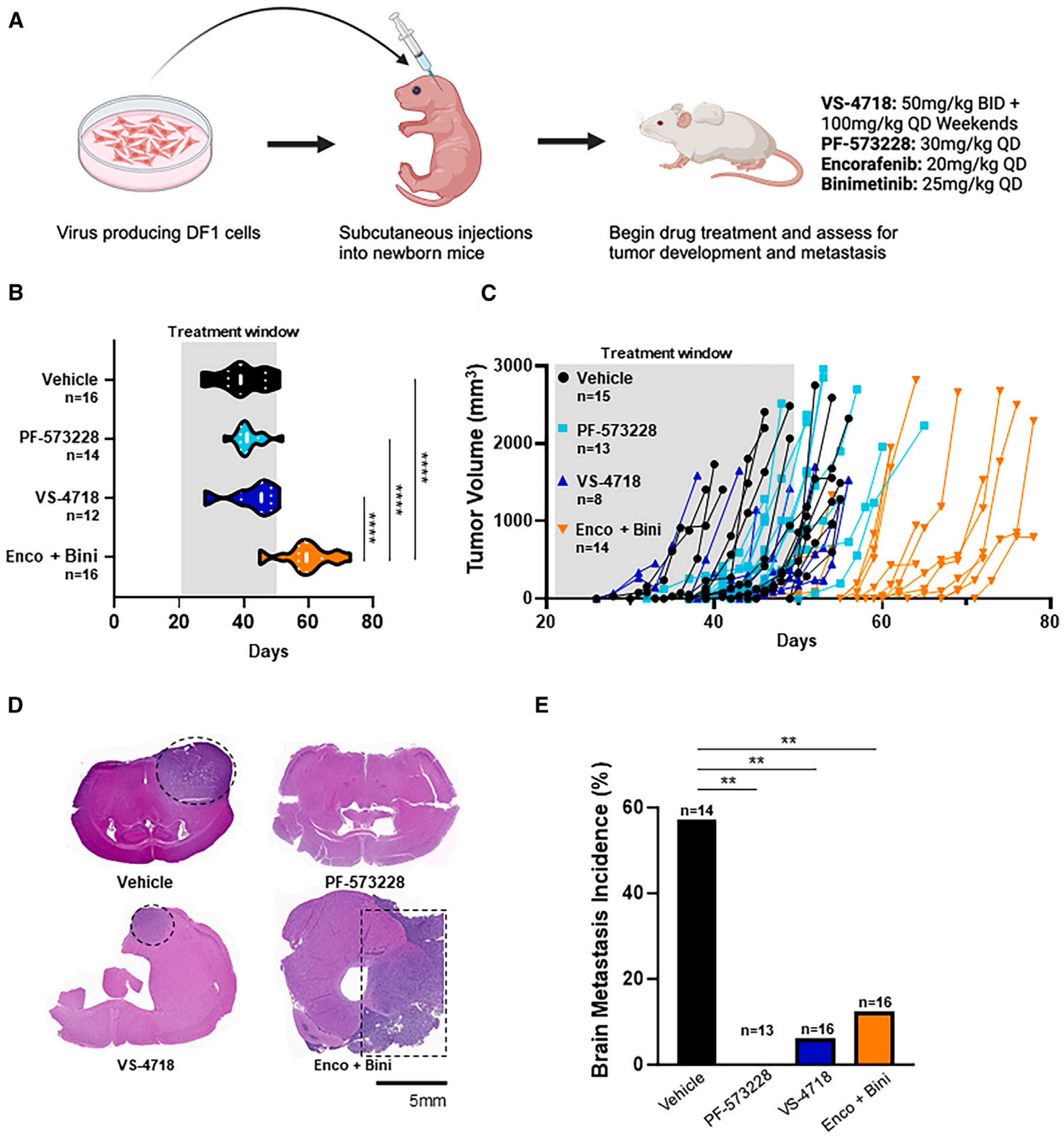

Figure 3. Pharmacological inhibition of FAK reduces the development of brain metastases from primary melanoma tumors

(A) Schematic of tumor induction and drug treatment in the RCAS/TVA mouse model. Created with BioRender.com.

(B) Violin plot illustrating tumor onset for *Dct::TVA;Braf*^{CA}*;Cdkn2a*$^{lox/lox}$*;Pten*$^{lox/lox}$ newborn mice injected with DF-1 fibroblasts harboring RCAS-Cre and RCAS-myrAKT1 and randomized into cohorts treated with vehicle (black), PF-573228 (30 mg/kg orally [PO] once daily [QD]; cyan), VS-4718 (50 mg/kg PO twice daily [BID]; blue), and encorafenib in combination with binimetinib (Enco + Bini; 20 mg/kg and 25 mg/kg, respectively, PO QD; orange). Mice were treated for 28 days, and tumor onset was tracked. A one-way ANOVA was used to determine statistical significance. Mouse numbers in each cohort are indicated on the y axis. Mean values +/− top and bottom quartiles are denoted by dotted lines.

(C) Individual tumor growth trajectories over time beginning on the day of injection. Mouse numbers in each cohort are indicated below each treatment.

(D) Brains of mice were examined histologically for metastases. Representative H&E images are shown for each cohort. Melanoma brain metastases are demarcated with a dashed line if present. Scale bar represents 5 mm.

(E) Bar graph showing percent incidence of brain metastases for the cohorts indicated. A Fisher's exact test was used to determine significance. Mouse numbers in each cohort are indicated above each bar. p value is $p < 0.01$ (**). Total mouse numbers and sex of each are as follows: vehicle $n = 16$ (10 females and 6 males), PF-573228 $n = 15$ (5 females and 10 males), VS-4718 $n = 12$ (5 females and 7 males), and Enco + Bini $n = 16$ (8 females and 8 males).

The number of mice differs across panels, as not all subjects were evaluable in each experimental condition.

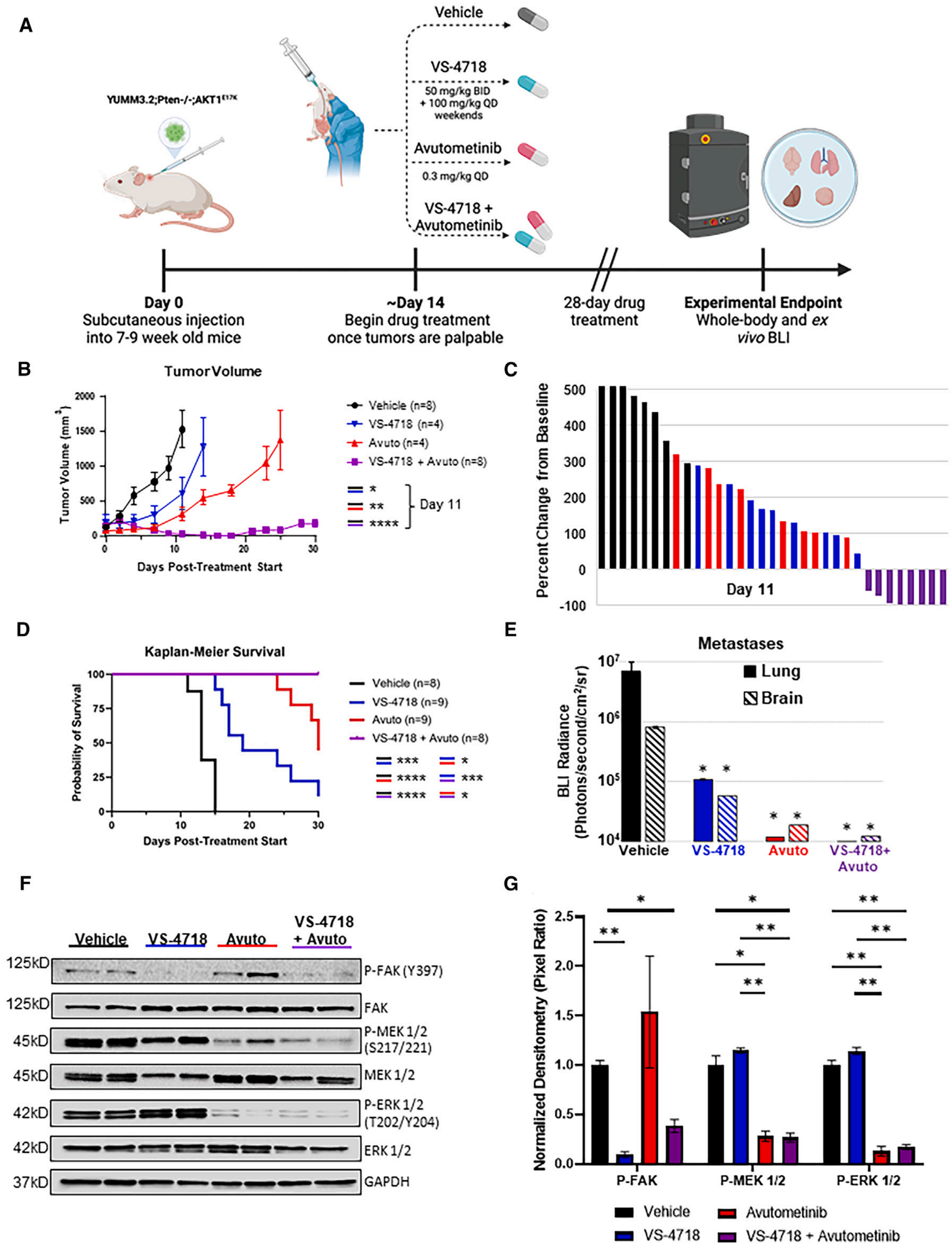

Figure 4. Combined FAK/RAF/MEK inhibition significantly reduces tumor growth, prolongs overall survival, and reduces metastases in mice with established primary melanoma tumors

(A) Schematic of subcutaneous tumor induction, BLI, and drug treatment in the syngeneic melanoma mouse model. Created with BioRender.com.

(B) Mean tumor volume over time. C57BL/6 glowing head mice were transplanted subcutaneously with luciferase-expressing YUMM3.2;Pten$^{-/-}$;AKT1^{E17K} cells. Once tumors were measurable, mice were randomized into vehicle (black), VS-4718 (50 mg/kg PO BID; blue), avutometinib (0.3 mg/kg PO QD; red), or VS-4718

(legend continued on next page)

options. Utilizing preclinical mouse models that recapitulate the genetic alterations most commonly found in human melanoma including BRAF^{V600E} along with loss of *PTEN* and *CDKN2A*, we previously demonstrated that hyperactivation of AKT1 promotes melanoma invasion and metastasis, particularly to the lungs and brain.[5,27] This observation is consistent with previous studies showing increased P-AKT and decreased PTEN levels in human melanoma brain metastases.[2–4] Although the FDA recently approved the AKT inhibitor capivasertib in combination with the estrogen receptor antagonist fulvestrant for the treatment of hormone receptor (HR)-positive, human epidermal growth factor receptor 2 (HER2)-negative advanced or metastatic breast cancer in patients whose tumors harbor alterations in PIK3CA, AKT1, or PTEN,[29] clinical trials investigating pharmacological AKT inhibitors in melanoma have shown limited efficacy.[6,7] Our prior work provides insight into the differential responses of melanoma cells to pharmacological versus genetic AKT inhibition. While pharmacological AKT inhibition failed to induce significant melanoma cell death *in vitro* or *in vivo*, genetic silencing of AKT resulted in profound melanoma cell death. This discrepancy can be attributed to the relief of negative feedback signaling upon pharmacological inhibition, leading to paradoxical pathway activation, which was effectively overcome by genetic silencing of AKT.[8] Given the lack of efficacy of AKT inhibitors in melanoma, we used our established mouse models to further elucidate the molecular mechanisms by which AKT1 contributes to metastatic spread in an effort to identify alternative therapeutic targets. This approach revealed an AKT1-specific role in upregulating FA signaling through phosphorylation of FAK.[5]

In this study, we utilized our mouse models to further explore the therapeutic potential of FAK inhibition either alone or in combination with RAF/MEK blockade, for the prevention and treatment of melanoma metastases. FAK, a key regulator of cancer cell motility and invasion, has emerged as a promising target for multiple malignancies. Our *in vitro* studies demonstrate that FAK inhibition synergizes with RAF/MEK blockade to suppress melanoma cell proliferation, highlighting the therapeutic potential of combining these agents. Importantly, our *in vivo* experiments validated these findings, showing that combined FAK/RAF/MEK inhibition not only inhibited primary tumor growth but also significantly reduced the incidence of lung and brain metastases and prolonged overall survival in mouse models.

Furthermore, our study provides insight into the crosstalk between FAK and MAPK signaling pathways. Prior studies have demonstrated that the MAPK pathway can exert negative regulation on FAK signaling; in fibroblasts expressing mutant RAS, ERK-mediated phosphorylation of FAK at Serine 910 (S910) triggered a feedback mechanism leading to FAK inactivation through dephosphorylation and FA turnover during migration.[30] We observed reciprocal activation of FAK following treatment with avutometinib or encorafenib, and the loss of this negative regulation might, in part, elucidate the activation of FAK in cancer cells subsequent to MAPK pathway blockade. Combined inhibition of FAK and RAF/MEK tempered this feedback activation, resulting in enhanced therapeutic efficacy.

To uncover synthetic lethal gene interactions in the context of FAK inhibition in G protein subunit alpha q (GNAQ)-mutant uveal melanoma (UM), Paradis et al. utilized a kinome-wide CRISPR-Cas9 screen. The genomic profile of UM differs notably from other melanomas and is characterized by tumor initiating GNAQ/11 mutations. While canonical GNAQ/11 signaling involves phospholipase C beta (PLCβ)-protein kinase C (PKC), the authors revealed a non-canonical pathway involving Hippo/yes-associated protein (YAP) activation via FAK, leading to extracellular signal-regulated kinase (ERK)-mediated resistance to FAK inhibition. Their study showcased potent synergy between MEK and FAKi, reducing proliferation and enhancing apoptosis both *in vitro* and *in vivo*. Paradis and colleagues' findings further our understanding of GNAQ/11 signaling and propose a promising combination therapy approach, necessitating further exploration.[31] The recent study by Tarin et al. builds upon these findings by investigating the combined impact of FAK inhibition with various drugs targeting known UM-related pathways across multiple cell lines. They found that inhibiting both FAK and MEK or PKC together had synergistic effects, decreasing cell viability and inducing apoptosis. Additionally, these combinations showed promising results in UM patient-derived xenografts, indicating their potential as a therapeutic strategy for metastatic UM.[32]

The research discussed does not exclude the potential benefits of targeting pathways that extend further from MAPK-centric signaling. Notably, we observed that total FAK protein levels in YUMM3.2 cells increased following treatment with VS-4718 (Figures 1E and 1G), indicating an adaptive response aimed at re-establishing FAK signaling. One possible mechanism for this

and avutometinib (purple) cohorts. A one-way ANOVA was used to determine statistical significance in tumor volume on day 11. Mean values +/− SEM are reported. Mouse numbers in each cohort are indicated in the legend. p values are as follows: $p < 0.05$ (*), $p < 0.01$ (**), $p < 0.0001$ (****).

(C) Waterfall plot depicting the tumor objective response rate from (B) at day 11. Each bar represents an individual mouse.

(D) Kaplan-Meier percent survival curves for mice treated with vehicle (black), VS-4718 (blue), avutometinib (red), or VS-4718 and avutometinib (purple). A log rank (Mantel Cox) test was used to determine statistical significance. Mouse numbers in each cohort are indicated in the legend. p values are as follows: $p < 0.05$ (*), $p < 0.01$ (**), $p < 0.001$ (***), $p < 0.0001$ (****); ns, not significant.

(E) Bioluminescence imaging (BLI) on the lungs and brains of mice in (D) was performed *ex vivo*, following the injection of luciferin. A Tukey's multiple comparison test was used to determine statistical significance in BLI radiance (photons/second/cm^2/steradian [sr]). p value is as follows: $p < 0.05$ (*).

(F) Immunoblot analyses were performed using primary tumor lysates and antibodies against P-FAK (Y397), FAK, P-MEK1/2 (S217/221), MEK1/2, and GAPDH (loading control).

(G) Quantitation of immunoblot data in (F) performed in duplicate. P-FAK was normalized to total FAK, P-MEK1/2 was normalized to total MEK1/2, and P-ERK1/2 was normalized to total ERK1/2. Values are expressed relative to vehicle control. p values are as follows: $p < 0.05$ (*), $p < 0.01$ (**). Total mouse numbers and sex of each are as follows: vehicle $n = 11$ (4 females and 7 males), VS-4718 $n = 14$ (7 females and 7 males), avutometinib $n = 9$ (3 females and 6 males), and VS-4718 and avutometinib $n = 10$ (5 females and 5 males).

The number of mice differs across panels, as not all subjects were evaluable in each experimental condition. See also Figure S3.

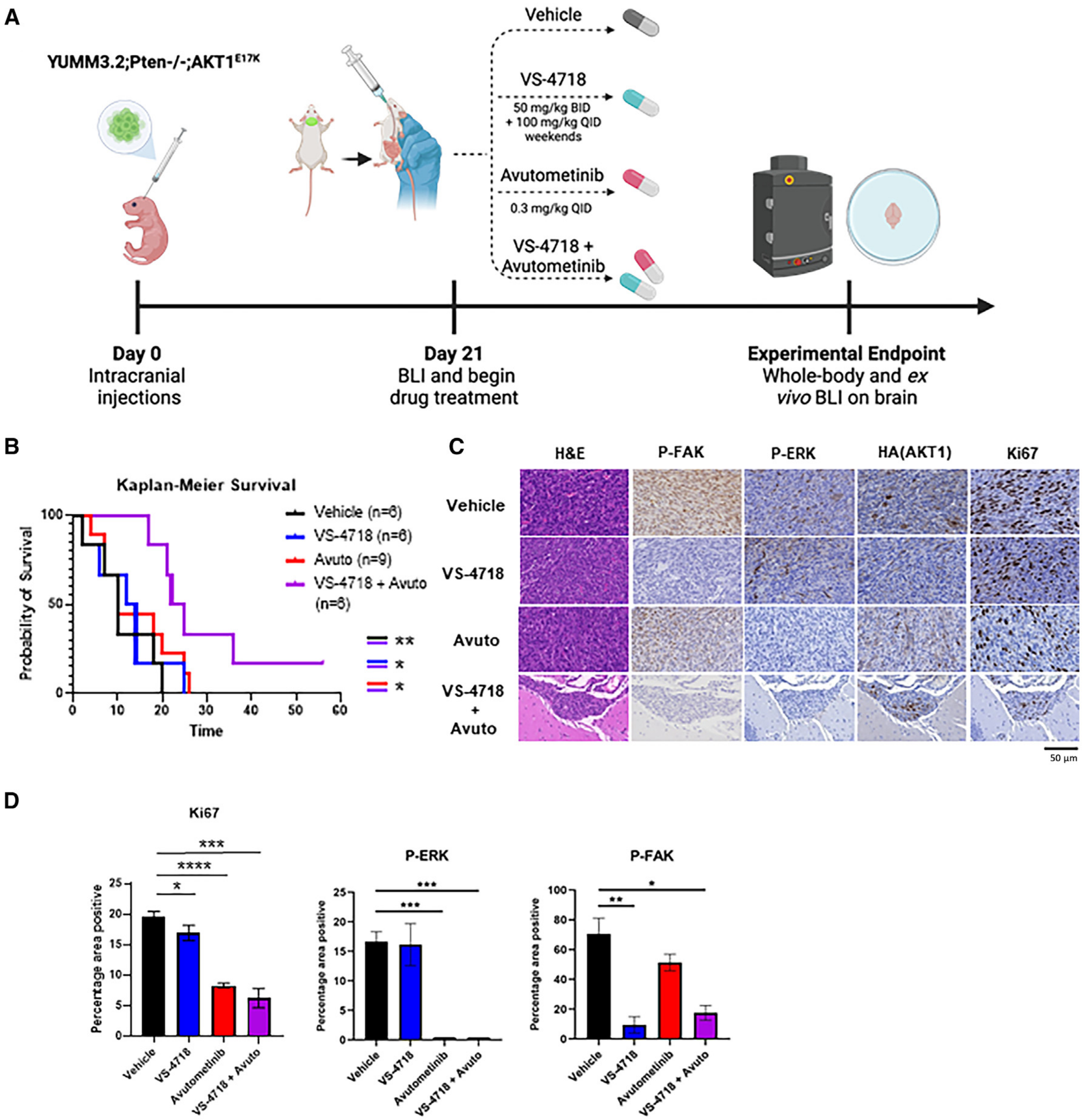

Figure 5. Combined FAK/RAF/MEK inhibition significantly prolongs survival in mice with existing brain metastases

(A) Schematic of intracranial tumor induction, BLI, and drug treatment in the C57BL/6 syngeneic mouse model. Created with BioRender.com.

(B) Kaplan-Meier percent survival curves for newborn glowing head mice intracranially injected with luciferase-expressing YUMM3.2;Pten$^{-/-}$;AKT1^{E17K} cells and treated with vehicle (black), VS-4718 (50 mg/kg PO BID; blue), avutometinib (0.3 mg/kg PO QD; red), or VS-4718 and avutometinib (purple) for 28 days upon weaning. A log rank (Mantel Cox) test was used to determine statistical differences. Mouse numbers in each cohort are indicated in the legend. p values are as follows: $p < 0.05$ (*), $p < 0.01$ (**).

(C) Histological analyses were performed on the brain sections of mice. Sections were stained for H&E, and immunohistochemistry performed using antibodies against Ki67, P-FAK, P-ERK1/2, and HA (AKT1^{E17K}); representative images of tumor regions are shown (left to right); scale bar, 50 μm.

(D) Ki67, P-ERK, and P-FAK quantification in tumors. Three high power fields were evaluated for each sample. An unpaired t test was used to determine statistical significance. p values are as follows: $p < 0.05$ (*), $p < 0.001$ (***), $p < 0.0001$ (****). Total mouse numbers and sex of each are as follows: vehicle $n = 6$ (3 females and 3 males), VS-4718 $n = 6$ (1 female and 5 males), avutometinib $n = 9$ (5 females and 4 males), and VS-4718 and avutometinib $n = 6$ (1 female and 5 males). See also Figure S4.

increase is the activation of transcription factors such as YAP and transcriptional co-activator with PDZ-binding motif (TAZ), which respond to changes in mechanotransduction and can up-regulate FAK expression in contexts of diminished FAK activity.[33] This suggests that combining FAKi with agents that target YAP/TAZ or nuclear factor κB may reduce the transcriptional up-regulation of FAK, thereby enhancing the efficacy of FAK-targeted therapies. Considering the role of YAP as an activator of

FAK, which promotes ERK-mediated resistance to FAKi,[31] YAP could be an attractive target that warrants further investigation in this context. Understanding these adaptive mechanisms is essential for developing effective therapeutic strategies against CM that prevent the rebound of FAK signaling.

The current standard of care for patients with mutant BRAF melanoma who have failed immunotherapy is the combined inhibition of BRAF and MEK. To identify mechanisms of resistance to combined inhibition of mutant BRAF and MEK in CM, Lubrano et al. interrogated the transcriptomes of pre- and post-treatment biopsies from patients who developed resistance to this treatment regimen and found activation of FA signaling pathways in post-treatment tumor samples. Consequently, inhibiting FAK alongside RAF/MEK with avutometinib showed promise in overcoming this resistance, demonstrating synergistic effects in inhibiting cell proliferation and promoting cell death.[34] These findings in melanomas resistant to MAPK pathway blockade, as well as our results from this study, suggest a potential avenue not only for FAK/RAF/MEK inhibition in combination in patients with $BRAF^{V600E}$-driven CM who have progressed on targeted therapy but also for those with brain metastases or at risk for developing brain metastases. In addition, since avutometinib binds MEK and locks the protein in a complex with RAF, this strategy should also be effective in melanomas driven by mutant NRAS or loss of *neurofibromin* (*NF1*). In accordance with this, we are currently testing the clinical efficacy of defactinib and avutometinib in patients with brain metastases from all molecular subtypes of CM who have failed either first-line immunotherapy or both first-line immunotherapy and second-line targeted therapy (phase 1b/2 trial of defactinib and avutometinib, with or without encorafenib, for the treatment of patients with brain metastases from cutaneous melanoma [DETERMINE]; NCT06194929). The promising outcomes of combined FAK/RAF/MEK inhibition not only offer hope for overcoming resistance in $BRAF^{V600E}$-driven CM but also signify a potential breakthrough for patients with brain metastases, underscoring the urgency of further clinical exploration.

Limitations of the study

We did not determine whether tumor regression and reduced brain metastases are linked to tumor cell-intrinsic mechanisms, immune-mediated responses, or a combination of both. While this study makes significant strides in understanding the effects of FAK and RAF/MEK inhibition in melanoma models driven by mutant BRAF, it is possible that our findings may not represent the biology of all melanoma patients with brain metastases including those driven by mutant NRAS or loss of NF1. Moreover, the reliance on preclinical mouse models introduces limitations in direct applicability to clinical outcomes. Further exploration through clinical trials is necessary to confirm the efficacy of the proposed therapeutic combinations in patients.

RESOURCE AVAILABILITY

Lead contact

Further information and requests for resources and reagents used in this study are available with a completed materials transfer agreement from the lead contact, Dr. Sheri L. Holmen (sheri.holmen@hci.utah.edu).

Materials availability

This study did not generate new unique reagents.

Data and code availability

All raw data are available upon request. This paper does not report original code. The software used in this study is described in the aforementioned section and the key resources table in detail. Any additional information required to reanalyze the data reported in this paper is available from the lead contact upon request.

ACKNOWLEDGMENTS

We thank members of the M.V., M.M., A.W., and S.H. labs for providing mouse strains, reagents, vectors, and advice. We especially thank S.S. for his technical assistance. We thank Verastem Oncology for generously providing VS-4718 and avutometinib. Research reported in this publication utilized the Research Informatics, Flow Cytometry, Histology, and DNA Sequencing cores as well as the Preclinical Research Resource at Huntsman Cancer Institute at the University of Utah. These core facilities and shared resources are supported by the National Cancer Institute of the National Institutes of Health (NIH) under award number P30CA042014. The Flow Cytometry core is also supported by the Office of the Director, NIH, under award number S10OD026959. J.A., G.L.P., K.A.S., and S.L.H. were supported by the Huntsman Cancer Foundation and grants from the NIH (T32TR004394, F31CA254307, T32CA265782, and R01CA121118, respectively). The content is solely the responsibility of the authors and does not necessarily represent the official views of the NIH.

AUTHOR CONTRIBUTIONS

J.A., T.T., K.A.S., D.A.K., H.C., S.C., J.A.P., and S.L.H. designed experiments. J.A., T.T., K.A.S., K.C., D.A.K., M.N.F., and G.L.P. performed experiments. J.A., T.T., K.A.S., D.A.K., and S.L.H. analyzed the data. J.A., T.T., D.A.K., and S.L.H. wrote the manuscript. All authors discussed the results and revised the manuscript accordingly.

DECLARATION OF INTERESTS

S.C. and J.A.P. are employees and stockholders of Verastem Oncology (Needham, MA).

STAR★METHODS

Detailed methods are provided in the online version of this paper and include the following:

- KEY RESOURCES TABLE
  - Contact for reagent and resource sharing
- EXPERIMENTAL MODEL AND STUDY PARTICIPATION DETAILS
  - Mice and genotyping
  - Viral constructs and virus production
  - Stable cell line generation
  - Cell culture
  - *In vivo* viral infections and RCAS tumor induction
  - *In vivo* syngeneic allografts
  - *In vivo* intracranial model
  - *In vivo* drug treatments
- METHOD DETAILS
  - Inhibitors
  - Incucyte assays
  - Synergy analysis
- BIOLUMINESCENCE IMAGING
  - Histology and histochemical staining
  - Immunohistochemistry
  - Immunoblotting
- QUANTIFICATION AND STATISTICAL ANALYSIS

CellPress
OPEN ACCESS

SUPPLEMENTAL INFORMATION

Supplemental information can be found online at https://doi.org/10.1016/j.xcrm.2025.101943.

Received: July 12, 2024
Revised: October 4, 2024
Accepted: January 13, 2025
Published: February 7, 2025

REFERENCES

1. Dohm, A.E., Nakashima, J.Y., Kalagotla, H., Jiang, S.X., Tang, J.D., Bhandari, M., Kim, Y., Graham, J.A., Khushalani, N.I., Forsyth, P.A., et al. (2023). Stereotactic radiosurgery and anti-PD-1 + CTLA-4 therapy, anti-PD-1 therapy, anti-CTLA-4 therapy, BRAF/MEK inhibitors, BRAF inhibitors, or conventional chemotherapy for the management of melanoma brain metastases. Eur. J. Cancer *192*, 113287. https://doi.org/10.1016/j.ejca.2023.113287.
2. Dai, D.L., Martinka, M., and Li, G. (2005). Prognostic Significance of Activated Akt Expression in Melanoma: A Clinicopathologic Study of 292 Cases. J. Clin. Oncol. *23*, 1473–1482. https://doi.org/10.1200/jco.2005.07.168.
3. Davies, M.A., Stemke-Hale, K., Lin, E., Tellez, C., Deng, W., Gopal, Y.N., Woodman, S.E., Calderone, T.C., Ju, Z., Lazar, A.J., et al. (2009). Integrated Molecular and Clinical Analysis of AKT Activation in Metastatic Melanoma. Clin. Cancer Res. *15*, 7538–7546. https://doi.org/10.1158/1078-0432.ccr-09-1985.
4. Stahl, J.M., Sharma, A., Cheung, M., Zimmerman, M., Cheng, J.Q., Bosenberg, M.W., Kester, M., Sandirasegarane, L., and Robertson, G.P. (2004). Deregulated Akt3 Activity Promotes Development of Malignant Melanoma. Cancer Res. *64*, 7002–7010. https://doi.org/10.1158/0008-5472.can-04-1399.
5. Kircher, D.A., Trombetti, K.A., Silvis, M.R., Parkman, G.L., Fischer, G.M., Angel, S.N., Stehn, C.M., Strain, S.C., Grossmann, A.H., Duffy, K.L., et al. (2019). AKT1(E17K) Activates Focal Adhesion Kinase and Promotes Melanoma Brain Metastasis. Mol. Cancer Res. *17*, 1787–1800. https://doi.org/10.1158/1541-7786.MCR-18-1372.
6. Algazi, A.P., Esteve-Puig, R., Nosrati, A., Hinds, B., Hobbs-Muthukumar, A., Nandoskar, P., Ortiz-Urda, S., Chapman, P.B., and Daud, A. (2018). Dual MEK/AKT inhibition with trametinib and GSK2141795 does not yield clinical benefit in metastatic NRAS-mutant and wild-type melanoma. Pigment Cell Melanoma Res. *31*, 110–114. https://doi.org/10.1111/pcmr.12644.
7. Algazi, A.P., Moon, J., Lao, C.D., Chmielowski, B., Kendra, K.L., Lewis, K.D., Gonzalez, R., Kim, K., Godwin, J.E., Curti, B.D., et al. (2024). A phase 1 study of triple-targeted therapy with BRAF, MEK, and AKT inhibitors for patients with BRAF-mutated cancers. Cancer *130*, 1784–1796. https://doi.org/10.1002/cncr.35200.
8. Parkman, G.L., Turapov, T., Kircher, D.A., Burnett, W.J., Stehn, C.M., O'Toole, K., Culver, K.M., Chadwick, A.T., Elmer, R.C., Flaherty, R., et al. (2024). Genetic Silencing of AKT Induces Melanoma Cell Death via mTOR Suppression. Mol. Cancer Ther. *23*, 301–315. https://doi.org/10.1158/1535-7163.MCT-23-0474.
9. Chuang, H.H., Zhen, Y.Y., Tsai, Y.C., Chuang, C.H., Hsiao, M., Huang, M.S., and Yang, C.J. (2022). FAK in Cancer: From Mechanisms to Therapeutic Strategies. Int. J. Mol. Sci. *23*, 1726. https://doi.org/10.3390/ijms23031726.
10. Chen, G., Gao, C., Gao, X., Zhang, D.H., Kuan, S.F., Burns, T.F., and Hu, J. (2018). Wnt/beta-Catenin Pathway Activation Mediates Adaptive Resistance to BRAF Inhibition in Colorectal Cancer. Mol. Cancer Ther. *17*, 806–813. https://doi.org/10.1158/1535-7163.MCT-17-0561.
11. Hirata, E., Girotti, M.R., Viros, A., Hooper, S., Spencer-Dene, B., Matsuda, M., Larkin, J., Marais, R., and Sahai, E. (2015). Intravital imaging reveals how BRAF inhibition generates drug-tolerant microenvironments with high integrin beta1/FAK signaling. Cancer Cell *27*, 574–588. https://doi.org/10.1016/j.ccell.2015.03.008.
12. McNamara, B., Demirkiran, C., Hartwich, T.M.P., Bellone, S., Manavella, D., Mutlu, L., Greenman, M., Zipponi, M., Yang-Hartwich, Y., Yang, K., et al. (2024). Preclinical efficacy of RAF/MEK clamp avutometinib in combination with FAK inhibition in low grade serous ovarian cancer. Gynecol. Oncol. *183*, 133–140. https://doi.org/10.1016/j.ygyno.2024.01.028.
13. Pang, X.J., Liu, X.J., Liu, Y., Liu, W.B., Li, Y.R., Yu, G.X., Tian, X.Y., Zhang, Y.B., Song, J., Jin, C.Y., and Zhang, S.Y. (2021). Drug Discovery Targeting Focal Adhesion Kinase (FAK) as a Promising Cancer Therapy. Molecules *26*, 4250. https://doi.org/10.3390/molecules26144250.
14. Shapiro, I.M., Kolev, V.N., Vidal, C.M., Kadariya, Y., Ring, J.E., Wright, Q., Weaver, D.T., Menges, C., Padval, M., McClatchey, A.I., et al. (2014). Merlin deficiency predicts FAK inhibitor sensitivity: a synthetic lethal relationship. Sci. Transl. Med. *6*, 237ra68. https://doi.org/10.1126/scitranslmed.3008639.
15. Yoshimura, A., Horinaka, M., Yaoi, T., Ono, H., Itoh, K., Yamada, T., Takayama, K., and Sakai, T. (2024). Epithelial-mesenchymal transition status is a remarkable biomarker for the combination treatment with avutometinib and defactinib in KRAS-mutated non-small cell lung cancer. Br. J. Cancer *131*, 361–371. https://doi.org/10.1038/s41416-024-02727-2.
16. Hartwich, T.M.P., Mansolf, M., Demirkiran, C., Greenman, M., Bellone, S., McNamara, B., Nandi, S.P., Alexandrov, L.B., Yang-Hartwich, Y., Coma, S., et al. (2024). Preclinical evaluation of avutometinib and defactinib in high-grade endometrioid endometrial cancer. Cancer Med. *13*, e70210. https://doi.org/10.1002/cam4.70210.
17. Demirkiran, C., Greenman, M., Bellone, S., McNamara, B., Hartwich, T.M.P., Manavella, D., Mutlu, L., Zipponi, M., Yang-Hartwich, Y., Yang, K., et al. (2024). Preclinical in vitro and in vivo activity of the RAF/MEK clamp avutometinib in combination with FAK inhibition in uterine carcinosarcomas. Gynecol. Oncol. *187*, 12–20. https://doi.org/10.1016/j.ygyno.2024.04.010.
18. Dawson, J.C., Serrels, A., Stupack, D.G., Schlaepfer, D.D., and Frame, M.C. (2021). Targeting FAK in anticancer combination therapies. Nat. Rev. Cancer *21*, 313–324. https://doi.org/10.1038/s41568-021-00340-6.
19. Gerber, D.E., Camidge, D.R., Morgensztern, D., Cetnar, J., Kelly, R.J., Ramalingam, S.S., Spigel, D.R., Jeong, W., Scaglioni, P.P., Zhang, S., et al. (2020). Phase 2 study of the focal adhesion kinase inhibitor defactinib (VS-6063) in previously treated advanced KRAS mutant non-small cell lung cancer. Lung Cancer *139*, 60–67. https://doi.org/10.1016/j.lungcan.2019.10.033.
20. Banerjee, S.N., Ring, K.L., Van Nieuwenhuysen, E., Fabbro, M., Aghajanian, C., Oaknin, A., Colombo, N., Santin, A., Clamp, A.R., Moore, K.N., et al. (2023). Initial efficacy and safety results from ENGOT-ov60/GOG-3052/RAMP 201: A phase 2 study of avutometinib (VS-6766) ± defactinib in recurrent low-grade serous ovarian cancer (LGSOC). J. Clin. Oncol. *41*, 5515. https://doi.org/10.1200/JCO.2023.41.16_suppl.5515.
21. Wang-Gillam, A., Lim, K.H., McWilliams, R., Suresh, R., Lockhart, A.C., Brown, A., Breden, M., Belle, J.I., Herndon, J., Bogner, S.J., et al. (2022). Defactinib, Pembrolizumab, and Gemcitabine in Patients with Advanced Treatment Refractory Pancreatic Cancer: A Phase I Dose Escalation and Expansion Study. Clin. Cancer Res. *28*, 5254–5262. https://doi.org/10.1158/1078-0432.CCR-22-0308.
22. Ishii, N., Harada, N., Joseph, E.W., Ohara, K., Miura, T., Sakamoto, H., Matsuda, Y., Tomii, Y., Tachibana-Kondo, Y., Iikura, H., et al. (2013). Enhanced inhibition of ERK signaling by a novel allosteric MEK inhibitor, CH5126766, that suppresses feedback reactivation of RAF activity. Cancer Res. *73*, 4050–4060. https://doi.org/10.1158/0008-5472.CAN-12-3937.
23. Lito, P., Saborowski, A., Yue, J., Solomon, M., Joseph, E., Gadal, S., Saborowski, M., Kastenhuber, E., Fellmann, C., Ohara, K., et al. (2014). Disruption of CRAF-mediated MEK activation is required for effective MEK inhibition in KRAS mutant tumors. Cancer Cell *25*, 697–710. https://doi.org/10.1016/j.ccr.2014.03.011.

24. Banerjee, S.N., Monk, B.J., Van Nieuwenhuysen, E., Moore, K.N., Oaknin, A., Fabbro, M., Colombo, N., O'Malley, D.M., Coleman, R.L., Oza, A.M., et al. (2022). ENGOT-ov60/GOG-3052/RAMP 201: A phase 2 study of VS-6766 (RAF/MEK clamp) alone and in combination with defactinib (FAK inhibitor) in recurrent low-grade serous ovarian cancer (LGSOC). J. Clin. Oncol. *40*, TPS5615. https://doi.org/10.1200/JCO.2022.40.16_suppl.TPS5615.

25. Meeth, K., Wang, J.X., Micevic, G., Damsky, W., and Bosenberg, M.W. (2016). The YUMM lines: a series of congenic mouse melanoma cell lines with defined genetic alterations. Pigment Cell Melanoma Res. *29*, 590–597. https://doi.org/10.1111/pcmr.12498.

26. Duarte, D., and Vale, N. (2022). Evaluation of synergism in drug combinations and reference models for future orientations in oncology. Curr. Res. Pharmacol. Drug Discov. *3*, 100110. https://doi.org/10.1016/j.crphar.2022.100110.

27. Cho, J.H., Robinson, J.P., Arave, R.A., Burnett, W.J., Kircher, D.A., Chen, G., Davies, M.A., Grossmann, A.H., VanBrocklin, M.W., McMahon, M., and Holmen, S.L. (2015). AKT1 Activation Promotes Development of Melanoma Metastases. Cell Rep. *13*, 898–905. https://doi.org/10.1016/j.celrep.2015.09.057.

28. Day, C.P., Carter, J., Weaver Ohler, Z., Bonomi, C., El Meskini, R., Martin, P., Graff-Cherry, C., Feigenbaum, L., Tüting, T., Van Dyke, T., et al. (2014). Glowing head" mice: a genetic tool enabling reliable preclinical image-based evaluation of cancers in immunocompetent allografts. PLoS One *9*, e109956. https://doi.org/10.1371/journal.pone.0109956.

29. Mullard, A. (2024). FDA approves first-in-class AKT inhibitor. Nat. Rev. Drug Discov. *23*, 9. https://doi.org/10.1038/d41573-023-00202-w.

30. Zheng, Y., Xia, Y., Hawke, D., Halle, M., Tremblay, M.L., Gao, X., Zhou, X.Z., Aldape, K., Cobb, M.H., Xie, K., et al. (2009). FAK phosphorylation by ERK primes ras-induced tyrosine dephosphorylation of FAK mediated by PIN1 and PTP-PEST. Mol. Cell *35*, 11–25. https://doi.org/10.1016/j.molcel.2009.06.013.

31. Paradis, J.S., Acosta, M., Saddawi-Konefka, R., Kishore, A., Gomes, F., Arang, N., Tiago, M., Coma, S., Lubrano, S., Wu, X., et al. (2021). Synthetic Lethal Screens Reveal Cotargeting FAK and MEK as a Multimodal Precision Therapy for GNAQ-Driven Uveal Melanoma. Clin. Cancer Res. *27*, 3190–3200. https://doi.org/10.1158/1078-0432.CCR-20-3363.

32. Tarin, M., Nemati, F., Decaudin, D., Canbezdi, C., Marande, B., Silva, L., Derrien, H., Jochemsen, A.G., Gardrat, S., Piperno-Neumann, S., et al. (2023). FAK Inhibitor-Based Combinations with MEK or PKC Inhibitors Trigger Synergistic Antitumor Effects in Uveal Melanoma. Cancers *15*, 2280. https://doi.org/10.3390/cancers15082280.

33. Panciera, T., Azzolin, L., Cordenonsi, M., and Piccolo, S. (2017). Mechanobiology of YAP and TAZ in physiology and disease. Nat. Rev. Mol. Cell Biol. *18*, 758–770. https://doi.org/10.1038/nrm.2017.87.

34. Lubrano, S., Faraji, F., Cervantes-Villagrana, R.D., Ramirez, S., Arang, N., Officer, A., Rigiracciolo, D.C., Quiroz, P.Y.A., Bacchiocchi, A., Halaban, R., et al. (2024). Abstract 4745: A novel combination therapy targeting RAF, MEK and FAK to overcome skin cutaneous melanoma treatment resistance. Cancer Res. *84*, 4745. https://doi.org/10.1158/1538-7445.AM2024-4745

35. Bromberg-White, J.L., Webb, C.P., Patacsil, V.S., Miranti, C.K., Williams, B.O., and Holmen, S.L. (2004). Delivery of short hairpin RNA sequences by using a replication-competent avian retroviral vector. J. Virol. *78*, 4914–4916. https://doi.org/10.1128/jvi.78.9.4914-4916.2004.

36. VanBrocklin, M.W., Robinson, J.P., Lastwika, K.J., Khoury, J.D., and Holmen, S.L. (2010). Targeted delivery of NRASQ61R and Cre-recombinase to post-natal melanocytes induces melanoma in Ink4a/Arflox/lox mice. Pigment Cell Melanoma Res. *23*, 531–541. https://doi.org/10.1111/j.1755-148X.2010.00717.x.

37. Dankort, D., Filenova, E., Collado, M., Serrano, M., Jones, K., and McMahon, M. (2007). A new mouse model to explore the initiation, progression, and therapy of BRAFV600E-induced lung tumors. Genes Dev. *21*, 379–384. https://doi.org/10.1101/gad.1516407.

38. Zheng, H., Ying, H., Yan, H., Kimmelman, A.C., Hiller, D.J., Chen, A.J., Perry, S.R., Tonon, G., Chu, G.C., Ding, Z., et al. (2008). p53 and Pten control neural and glioma stem/progenitor cell renewal and differentiation. Nature *455*, 1129–1133. https://doi.org/10.1038/nature07443.

39. Davies, M.A., Stemke-Hale, K., Tellez, C., Calderone, T.L., Deng, W., Prieto, V.G., Lazar, A.J.F., Gershenwald, J.E., and Mills, G.B. (2008). A novel AKT3 mutation in melanoma tumours and cell lines. Br. J. Cancer *99*, 1265–1268.

STAR★METHODS

KEY RESOURCES TABLE

REAGENT or RESOURCE	SOURCE	IDENTIFIER
Antibodies		
Mouse anti-p27	ICL, Inc.	Cat# MALV-30A-6C2
Mouse anti-HA	Biolegend	Cat#901501; RRID: AB_2565005
Rabbit anti-HA	Cell Signaling Technology	Cat#3724; RRID:AB_1549585
Mouse anti-GAPDH	MilliporeSigma	Cat#MAB374; RRID: AB_2107445
Rabbit anti-Vinculin	Cell Signaling Technology	Cat#4650; RRID: AB_10559207
Rabbit anti-FAK	Cell Signaling Technology	Cat#3285; RRID: AB_2269034
Rabbit anti-P-FAK (Y397)	Cell Signaling Technology	Cat#3283; RRID: AB_2173659
Rabbit anti-P-FAK (Y925)	LSBio	Cat#LS-C177998
Rabbit anti-P-FAK (Y925)	Cell Signaling Technology	Cat#3284; RRID: AB_10831810
Rabbit anti-PTEN	Cell Signaling Technology	Cat#9188; RRID: AB_2253290
Rabbit anti-AKT	Cell Signaling Technology	Cat#4691; RRID: AB_915783
Rabbit anti-P-AKT (T308)	Cell Signaling Technology	Cat#13038; RRID: AB_2629447
Rabbit anti-P-AKT (S473)	Cell Signaling Technology	Cat#3787; RRID: AB_331170
Rabbit anti-PRAS40	Cell Signaling Technology	Cat#2691; RRID: AB_2225033
Rabbit anti-PRAS40 (T246)	Cell Signaling Technology	Cat#13175; RRID: AB_2798140
Rabbit anti-ERK1/2	Cell Signaling Technology	Cat#9107; RRID: AB_10695739
Rabbit anti-P-ERK1/2 (T202/Y204)	Cell Signaling Technology	Cat#4370; RRID: AB_2315112
Rabbit anti-MEK1/2	Cell Signaling Technology	Cat#9126; RRID: AB_331778
Rabbit anti-P-MEK1/2 (S217/S222)	Cell Signaling Technology	Cat#9154; RRID: AB_2138017
Rabbit anti-Cleaved Caspase-3 (D175)	Cell Signaling Technology	Cat#9661; RRID: AB_2341188
Rabbit anti-Ki67 (D3B5)	Cell Signaling Technology	Cat#12202; RRID: AB_2620142
Mouse anti-HRP conjugated	Cell Signaling Technology	Cat#7076; RRID: AB_330924
Rabbit anti-HRP conjugated	Cell Signaling Technology	Cat#7074; RRID: AB_2099233
Bacterial and virus strains		
Escherichia coli: F- mcrA Δ(mrr-hsdRMS-mcrBC) ϕ80lacZΔM15 ΔlacX74 recA1 araD139 Δ(ara- leu)7697 galU galK rpsL (StrR)	Thermo Fisher Scientific	One Shot^{TM} TOP10 Cat# C404010
Escherichia coli: F- mcrA Δ(mrr-hsdRMS-mcrBC) Φ80lacZΔM15 ΔlacX74 recA1 araΔ139 Δ(ara-leu)7697 galU galK rpsL (StrR)	Thermo Fisher Scientific	One Shot^{TM} *ccd*B survival^{TM} 2 T1^{R} Cat# A10460
Escherichia coli: F- ϕ80(lacZ)ΔM15 ΔlacX74 hsdR(rK-mK+) ΔrecA1398 endA1 tonA	Thermo Fisher Scientific	One Shot^{TM} Mach1^{TM} T1 Cat# C862003
Biological samples		N/A
Mouse tumor tissue	This paper	N/A
Mouse tail biopsies	This paper	N/A
Mouse brain tissue	This paper	N/A
Mouse lung tissue	This paper	N/A
Chemicals, peptides, and recombinant proteins		
PF-573228	Selleck Chemicals	Cat#S2013
Avutometinib	Verastem Oncology	N/A
VS-4718	Verastem Oncology	N/A
Encorafenib	Selleck Chemicals	Cat#S7108
Binimetinib	Selleck Chemicals	Cat#S7007

(*Continued on next page*)

Continued		
REAGENT or RESOURCE	SOURCE	IDENTIFIER
**Critical commercial assays**		
ATPLite Luminescence Assay Kit	Revvity	Cat#6016941
SignalStain DAB Substrate Kit	Cell Signaling Technology	Cat#8059
SignalStain Boost Detection Reagent	Cell Signaling Technology	Cat#8114 (Rabbit)<br>Cat#8125 (Mouse)
SignalStain Antibody Diluent	Cell Signaling Technology	Cat#8112
8-16% Tris-glycine polyacrylamide gel	Thermo Fisher Scientific	Cat#XP08160BOX (10-well)<br>Cat#XP08162BOX (12-well)
Enhanced chemiluminescence	Thermo Fisher Scientific	Cat#34580
**Deposited data**		
N/A		N/A
**Experimental models: Cell lines**		
293FT	Thermo Fisher Scientific	Cat#R70007
YUMM 3.2	Meeth et al.[25]	N/A
YUMM 3.2 (PTEN$^{-/-}$)	Parkman et al.[8]	N/A
YUMM3.2 (PTEN$^{-/-}$, HA-Akt1^{E17K})	This publication	N/A
YUMM3.2 (PTEN$^{-/-}$, HA-Akt1^{E17K}, Luciferase, eGFP)	This publication	N/A
DF-1	ATCC	Cat#CRL-3586
DF-1 (RCAS-Cre)	Cho et al.[27]	N/A
DF-1 (RCAS-myrAkt1)	Cho et al.[27]	N/A
**Experimental models: Organisms/strains**		
Dct::TVA;Braf^{CA};Cdkn2a$^{lox/lox}$;Pten$^{lox/lox}$	Cho et al.[27]	N/A
C57BL/6-*Tyr^{c-Brd}* Tg(Gh1-luc/EGFP)D8Mrln/J<br>C57BL/6 glowing head	Day et al.[28]<br>The Jackson Laboratory	Strain #027662
**Oligonucleotides**		
Primers for *TVA*<br>Fwd 5′-AGCTGGTGAGATGGGACTGAAC-3′<br>Rev 5′- CGAACATTCAAAGCCTCCAG-3′	IDT	N/A
Primers for *Braf^{CA}*<br>Fwd 5′-TGAGTATTTTTGTGGCAACTGC-3′<br>Rev 5′-CTCTGCTGGGAAAGCGGC-3′	IDT	N/A
Primers for *Pten*<br>CPF1: 5′CTTCGGAGCATGTCTGGCAATGC-3′<br>R1NEOCP: 5′CTGCACGAGACTAGTGAGACG TGC-3′<br>PTR14: 5′AAGGAAGAGGGTGGGGATAC-3′	IDT	N/A
Primers for *Cdkn2a*<br>Fwd 5′-TTGTTGGCCCAGGATGCCGACATC-3′<br>Rev 5′-CCAAGTGTGCAAACCCAGGCTCC-3′	IDT	N/A
**Recombinant DNA**		
pCR8 myr-HA-Akt1	Cho et al.[27]	N/A
pCR8 HA-Akt1^{E17K}	Kircher et al.[5]	N/A
pcDNA3.1-TVA	Bromberg-White et al.[35]	N/A
RCAS: replication-competent avian leukosis virus long terminal repeat with splice acceptor Bryan polymerase subgroup A	VanBrocklin et al.[36]	N/A
RCAS myr-HA-Akt1	Cho et al.[27]	N/A
RCAS HA-Akt1^{E17K}	Kircher et al.[5]	N/A
pDEST FG12-Luciferase-IRES-EGFP	Parkman et al.[8]	N/A
FG12-HA-AKT1^{E17K} Luciferase-IRES-EGFP	This publication	N/A
psPAX2	Addgene	Cat#12260
pCMV-VSV-G	Addgene	Cat#8454

(*Continued on next page*)

CellPress
OPEN ACCESS

Continued

REAGENT or RESOURCE	SOURCE	IDENTIFIER
Software and algorithms		
GraphPad Prism 7	Dotmatics	https://www.graphpad.com/scientific-software/prism/
R	Bell Laboratories	https://www.r-project.org/
ImageJ	ImageJ	https://imagej.net/ij/
Living Image	PerkinElmer	https://www.perkinelmer.com.cn/lab-products-and-services/resources/in-vivo-imaging-software-downloads.html?_ga=2.29909951.1041849854.1637318373-1604795059.1637318373#LivingImage
IncuCyte Analysis	Satorius	https://www.sartorius.com/en/products/live-cell-imaging-analysis/live-cell-analysis-software/
SynergyFinder	SynergyFinder	https://synergyfinder.fimm.fi/
Compusyn	ComboSyn	https://www.bioz.com/result/compusyn/
QuantCenter Image Analysis	3DHistech	https://www.3dhistech.com/research/quantcenter/

Contact for reagent and resource sharing

Further information and requests for resources and reagents should be directed to and will be fulfilled by the Lead Contact, Sheri L. Holmen (sheri.holmen@hci.utah.edu).

EXPERIMENTAL MODEL AND STUDY PARTICIPATION DETAILS

Mice and genotyping

All animal experimentation was performed in AAALAC approved facilities at the University of Utah. All animal protocols were reviewed and approved prior to experimentation by the Institutional Animal Care and Use Committee (IACUC) at the University of Utah. *Dct::TVA;Braf^{CA};Cdkn2a$^{lox/lox}$; Pten$^{lox/lox}$* mice were maintained on a mixed C57BL/6 and FVB/N background by random interbreeding. DNA from tail biopsies was used to genotype for the TVA transgene, *Braf^{CA}*, *Cdkn2a$^{lox/lox}$*, *Pten$^{lox/lox}$*, and wild-type alleles as described.[36–38] Both male and female newborn through adult mice were used in the RCAS/TVA study and in the C57BL/6 YUMM3.2 syngeneic studies. All mice were housed in cages containing up to five animals of the same sex and maintained at room temperature. Mice were fed a combination of Teklad Global 2920X and Teklad 3980X (Inotiv), supplemented with DietGel 76A and HydroGel (Clear H2O) post-weaning. One-week post-weaning, mice were transitioned to Teklad Global 2920X.

Viral constructs and virus production

The RCAS-Cre,[36] pCR8 myr-HA-Akt1 entry vector, pCR8 HA-Akt1^{E17K} entry vector, RCAS-myr-HA-Akt1, and RCAS-HA-Akt1^{E17K} constructs have been described.[5] DF-1 avian fibroblasts were transfected using calcium phosphate and RCAS-HA-Akt1^{E17K} proviral DNA as described.[5] Immunoblotting was used to monitor the expression of the p27 viral capsid protein using the anti-p27 antibody (ICL, Inc. Portland, OR; MALV-30A-6C2) and to confirm AKT1^{E17K} expression using the HA antibody (MMS-101P; Biolegend). Gateway cloning was used to recombine the pCR8 HA-Akt1^{E17K} entry clone and the pDEST FG12-Luciferase-IRES-EGFP lentiviral destination vector (Gene Universal) to generate the lentiviral vector FG12-HA-AKT1^{E17K} Luciferase-IRES-EGFP. Packaging plasmids psPAX2 (#12260, Addgene, Cambridge, MA) and pCMV-VSV-G (#8454, Addgene) were used in combination with FG12-HA-AKT1^{E17K} Luciferase-IRES-EGFP to generate virus for stable cell line generation as described below.

Stable cell line generation

Isogenic Yale University Mouse Melanoma 3.2 (YUMM3.2) PTEN^{WT} or $^{-/-}$ cells were transfected with pcDNA3.1-TVA[35] containing the Hygromycin B resistance gene to generate YUMM3.2-TVA positive cells. TVA-positive clones were selected for using 300 μg/mL Hygromycin B (Thermo Fisher). Supernatant from DF-1 cells producing RCAS-HA-AKT1^{E17K} was used to infect YUMM3.2-TVA^{+} cells. Expression of AKT1^{E17K} was confirmed by immunoblot (HA). A Pten-deficient isogenic variant of the YUMM 3.2 parental cell line was generated via CRISPR/CAS9.[8] To generate YUMM3.2 Pten$^{-/-}$ HA-Akt1^{E17K} Luciferase-EGFP cells, FG12-HA-AKT1^{E17K} Luciferase-IRES-EGFP, psPAX2, and pCMV-VSV-G vectors were transfected into 293FT cells using lipofectamine 3000 (Thermo Fisher). Supernatant from these cells containing virus was used to infect YUMM 3.2;PTEN^{WT} or $^{-/-}$ cells. Cells were sorted for EGFP using a Propel Labs Avalon cytometer at The Flow Cytometry Shared Resource Laboratory at the University of Utah. Expression of AKT1^{E17K} was confirmed by immunoblot (HA). DF-1 cells are available from ATCC (CRL-3586) and the sex is

unspecified. 293FT cells are available from Thermo Fisher Scientific (R70007) and the sex is unspecific. YUMM3.2 cells were a gift from Marcus Bosenberg and are derived from a female mouse melanoma. Cell line validation was performed using Short-Tandem Repeat (STR) genotyping at 24 loci.

Cell culture

DF-1 cells were grown in DMEM-high glucose media (Thermo Fisher) supplemented with 10% FBS (Atlas Biologicals), and 0.5 μg/mL gentamicin (Thermo Fisher), and maintained at 39°C in 5% CO_2. YUMM3.2 cells were grown in F12/DMEM media (Thermo Fisher) supplemented with 10% FBS, 1 μg/mL penicillin-streptomycin (Thermo Fisher), and 1 μg/mL non-essential amino acids (Thermo Fisher), and maintained at 37°C in 5% CO_2.

In vivo viral infections and RCAS tumor induction

Infected DF-1 cells from a confluent culture in a 10 cm dish were trypsinized, pelleted, resuspended in 100 μL of Hank's Balanced Salt Solution (HBSS) (Thermo Fisher), and placed on ice. Newborn mice were injected subcutaneously behind each ear with 50 μL of suspended DF-1 cells harboring RCAS-HA-myrAkt1 and RCAS-Cre. Mice were randomized into treatment arms and compounds were administered daily by oral gavage for 28 days when a primary tumor became palpable as described below (see *in vivo* drug treatment).

In vivo syngeneic allografts

Seven to nine-week-old C57BL/6 glowing head mice were injected subcutaneously into the dorsal area near the scapula with 5×10^4 YUMM3.2 $Pten^{-/-}$ cells co-expressing HA-$AKT1^{E17K}$, luciferase, and EGFP in matrigel and observed for tumor growth. Tumors were visualized and measured weekly using bioluminescence imaging (BLI), and tumor burden was quantified using bioluminescent photon output values and digital caliper measurements. The following formula was used to calculate tumor volume: (Length x $Width^2$)/2.

In vivo intracranial model

Newborn C57BL/6 glowing head mice were intracranially injected into the right cerebrum with 30 YUMM3.2 $Pten^{-/-}$ cells co-expressing HA-$AKT1^{E17K}$, luciferase, and EGFP, suspended in 5 μL of Hank's Balanced Salt Solution (HBSS) (Thermo Fisher) using a gas-tight Hamilton syringe. All mice were imaged with BLI upon weaning and weekly once treatment commenced.

In vivo drug treatments

For *in vivo* drug testing, drug treatment (or vehicle control treatment) was initiated at weaning (RCAS/TVA model), when tumors were measurable (*in vivo* syngeneic allografts), or once a BLI signal was detected (*in vivo* intracranial model). Mice were randomized based on tumor size and sex into vehicle, VS-4718 alone, PF-573228 alone, avutometinib alone, VS-4718 and avutometinib, or encorafenib and binimetinib treatment groups as indicated in each figure. VS-4718 was dosed via oral gavage at 50 mg/kg BID on weekdays and 100 mg/kg QD on weekends. PF-573228 was dosed via oral gavage at 30 mg/kg PO QD. Avutometinib was dosed via oral gavage at 0.3 mg/kg QD. Encorafenib was dosed via oral gavage at 20 mg/kg PO QD and binimetinib was dosed via oral gavage at 25 mg/kg PO QD. Vehicle for PF-573228, encorafenib and binimetinib was 0.5% carboxymethylcellulose with 0.5% Tween-80 in sterile H_2O. Vehicle for VS-4718 was 0.5% carboxymethylcellulose with 0.1% Tween-80 in sterile H_2O and vehicle for avutometinib was 10% Hydroxypropyl-B-Cyclodextrin +5% DMSO in sterile H_2O. The endpoint criteria were as follows: illness, ≥20% loss in body weight over a 7-day span, or the primary tumor reached 10% of total body weight. All mice were treated with a final drug dose 2 h prior to necropsy.

METHOD DETAILS

Inhibitors

The FAK inhibitor PF-573228, $BRAF^{V600E}$ inhibitor encorafenib, and MEK inhibitor binimetinib were purchased from Selleck Chemical. Avutometinib and VS-4718 were generously provided by Verastem Oncology. All drugs were formulated in DMSO for *in vitro* use and added to F12/DMEM media (Thermo Fisher) for a final concentration of 0.1% DMSO.

Incucyte assays

Cell proliferation was assessed by seeding ∼5,000 cells per well in 96-well plates. Experiments were performed three separate times in triplicate wells for each experimental condition. Pharmacological agents were added 24 h after plating. Cells were cultured in the presence or absence of pharmacological agents for 72 h. Confluence was assessed over time using an IncuCyte S3 Live Cell Imaging instrument with data analyzed using IncuCyte Analysis Software (Sartorius) at 2-h intervals.

Synergy analysis

Cells were plated in 96-well plates at 3,000 cells per well, and incubated at 37°C overnight. The following day, wells were treated with respective combinations of VS-4718 and avutometinib and incubated at 37°C for 72 h. Cell viability was analyzed using the ATPLite

Luminescence Assay Kit (PerkinElmer). Chou-Talalay combination index (CI) scores were calculated using Compusyn software whereas Bliss and Zip synergy scores were calculated using the SynergyFinder software.

BIOLUMINESCENCE IMAGING

Mice were injected intraperitoneally with 16.7 mg/mL D-Luciferin in 200μL of phosphate buffered saline 10 min prior to image acquisition. The IVIS Spectrum was used to acquire images at one-week intervals beginning 2 weeks after post-subcutaneous or intracranial implantation of mouse YUMM3.2 melanoma cells until the experimental endpoint. At sacrifice, the primary tumor as well as lungs and brain were imaged *ex vivo* using the IVIS Spectrum to confirm luciferase expression and to detect metastases. Living Image software (version 4.5.2) was used to compile images.

Histology and histochemical staining

Mice were euthanized at their experimental endpoints and subjected to a full necropsy. Brain, lung, and primary tumor tissues were fixed in formalin overnight, dehydrated in 70% ethyl alcohol, and paraffin embedded. Sections were stained with hematoxylin and eosin (H&E) or left unstained for immunohistochemistry (IHC).

Immunohistochemistry

Tissue sections from formalin-fixed paraffin embedded blocks were deparaffinized at 65 °C for 15 min, incubated in xylene, and rehydrated using decreasing concentrations of ethanol. Antigen retrieval was performed in a pressure cooker at 120°C for 30 min using citrate buffer (pH 6.0) for Akt1 (HA), P-ERK1/2, P-AKT, and Ki67, or EDTA (pH 8.0) for P-FAK. Peroxidase activity was quenched in 3% H_2O_2 for 10 min and slides were incubated in 5% normal goat serum in TBS-T (0.05% Tween 20) for 1 h inside a humidity chamber. Anti-rabbit primary antibodies were diluted in SignalStain Antibody Diluent (Cell Signaling Technology), added to slides, and incubated overnight at 4°C. Slides were incubated in SignalStain Boost Detection Reagent (Cell Signaling Technology) for 30 min in a humidity chamber and the SignalStain DAB Substrate Kit (Cell Signaling Technology) was used to detect the presence of each protein. Slides were counterstained in hematoxylin and dehydrated with increasing concentrations of alcohol and xylene prior to the addition of coverslips. Antibodies used and their concentrations include: HA (1:500; 3724 Cell Signaling Technology), P-ERK1/2 (T202/Y204) (1:400; 4370 Cell Signaling Technology), P-AKT (S473) (1:100; 3787 Cell Signaling Technology), Ki67 (1:400; 12202 Cell Signaling Technology), and P-FAK (Y925) (1:50; LS-C177998 LSBio). IHC quantification was performed using QuantCenter Image Analysis Software and ImageJ.

Immunoblotting

Cell lysates were suspended in 100 mM Tris-HCL, 4% SDS, 20% glycerol, and 10% DTT. To generate lysates from frozen primary tumors, tissues were pulverized in liquid nitrogen using a mortar and pestle and samples were resuspended in lysis buffer consisting of 50 mm HEPES (pH 7.4), 150 mmol/L NaCl, 1.5 mmol/L $MgCl_2$, 1 mmol/L EGTA, and 1% Triton X-100[39] with protease and phosphatase inhibitors (Pierce Biotechnology). All lysates were incubated at 95°C for 10 min, separated on an 8–16% Tris-glycine polyacrylamide gels (Thermo Fisher Scientific), and transferred to nitrocellulose for immunoblotting. Nitrocellulose membranes were incubated in blocking solution composed of 0.1% Tween 20 in 1X TBS with 5% nonfat dry milk for ALV p27, HA, GAPDH, and P-AKT (T308), or 5% BSA (Cell Signaling Technology) for all other antibodies. Blots were immunostained in the primary antibody diluted 1:1,000 (or 1:5,000 for GAPDH) in TBS-T for 1 h at room temperature or 4°C overnight with constant shaking and washed in TBS-T. Blots were then incubated in anti-mouse IgG-HRP or anti-rabbit IgG-HRP secondary antibody diluted 1:1,000 in TBS-T for 1 h with constant shaking and washed in TBS-T. Enhanced chemiluminescence (ECL, Amersham) was used according to the manufacturer's specifications and blots were imaged using the Azure Imaging system (Azure Biosystems). All antibodies are listed in resource table.

QUANTIFICATION AND STATISTICAL ANALYSIS

Cell proliferation assays were performed in triplicate. A Dunnett's multiple comparison test was used to determine statistical significance. Mean values +/− SEM are reported. Cell viability assays were performed in replicates of eight. A one-way ANOVA was used to determine statistical significance. Mean values +/− SD are reported. Brains of mice were examined histologically for metastases. A Fisher's exact test was used to determine statistical significance. Statistical differences in tumor onset were determined using a one-way ANOVA. Mean +/− top and bottom quartiles are reported. Statistical differences in tumor growth for Figure 4 were determined on the last day all mice were still on study (Day 11) using a one-way ANOVA. Mean ± SEM for tumor growth trajectories are reported over time. Statistical significance between Kaplan-Meier percent survival curves was determined using a Log rank (Mantel Cox) test. Statistical significance in bioluminescence radiance from the lungs and brains of mice was determined using a Tukey's multiple comparison test (Photons/second/cm^2/sr). Chou-Talalay (CI) synergy scores were determined using Compusyn software. Bliss and Zip synergy scores were determined using SynergyFinder software and are reported as mean ± SEM. Statistical significance in the weight change of mice was determined using a one-way ANOVA. Immunohistochemistry quantification was performed by random selection of three high-resolution 40X tissue sections in each cohort. The percentage of positivity for each target was

calculated based on the ratio of positive pixels to total pixels. Mean values +/− SEM are reported. Immunoblot quantification for cell proliferation assays were performed using ImageJ whereby a standard size region of interest was applied individually to all GAPDH protein bands to normalize targets of interest to GAPDH in all lanes. Post-GAPDH normalization, phosphoproteins were normalized to total protein for each respective target and a one-way ANOVA was used to determine statistical significance. For all experiments, the n of each treatment group or cohort (if not reported above) is reported in the figure or figure legend for each figure. All *p* values were determined in GraphPad Prism or R. The *p* values for all experiments are as follows: $p < 0.05$(*), $p < 0.01$(**), $p < 0.001$(***), $p < 0.0001$(****), ns = not significant.

Cell Reports Medicine, Volume *6*

Supplemental information

Combined inhibition of focal adhesion kinase and RAF/MEK elicits synergistic inhibition of melanoma growth and reduces metastases

Jared Almazan, Tursun Turapov, David A. Kircher, Karly A. Stanley, Katie Culver, A. Paulina Medellin, MiKaela N. Field, Gennie L. Parkman, Howard Colman, Silvia Coma, Jonathan A. Pachter, and Sheri L. Holmen

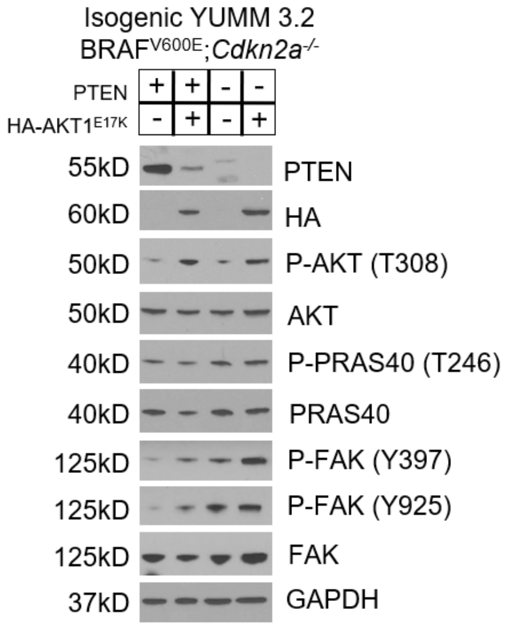

Supplemental Figure 1 title:

YUMM3.2 cells that express AKT^{E17K} and/or are deficient in PTEN show hyperactivity of the PI3K/AKT pathway *in vitro*, Related to Figure 1.

Supplemental Figure 1 legend:

Immunoblotting was used to quantify levels of PTEN, HA (AKT^{E17K}), P-AKT (T308), AKT, P-PRAS40 (T246), PRAS40, P-FAK (Y397, Y925), FAK, and GAPDH (loading control) in four isogenic YUMM3.2 cell lines +/- $AKT1^{E17K}$ and +/- PTEN.

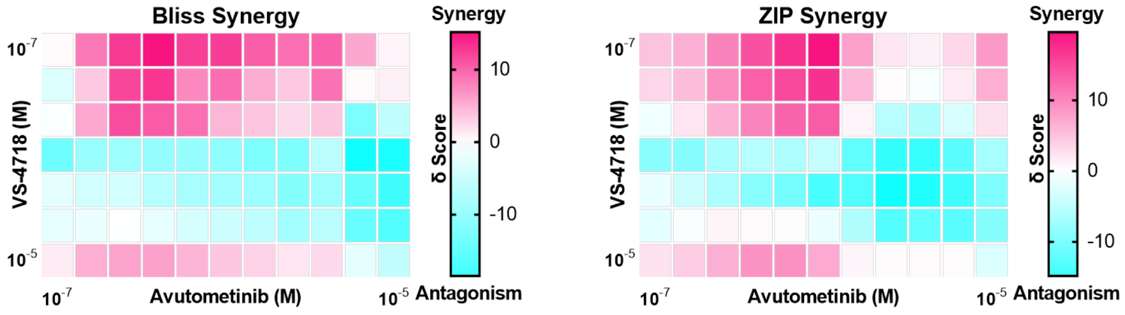

δ Score = 13.3 ± 2.4

δ Score = 13.8 ± 2.4

Supplemental Figure 2 title:

Combined FAK/RAF/MEK inhibition reduces YUMM3.2;Pten-/-;AKT1^{E17K} cell proliferation *in vitro*, Related to Figure 1.

Supplemental Figure 2 legend:

Cell viability was assessed in YUMM3.2;Pten-/-;AKT1^{E17K} cells treated with increasing concentrations of VS-4718 and avutometinib for 72 hours. Drug synergy was measured using Bliss and Zero Interaction Potency (Zip) tests with increasing concentrations of VS-4718 and avutometinib in combination; values greater than zero denote synergy.

A.

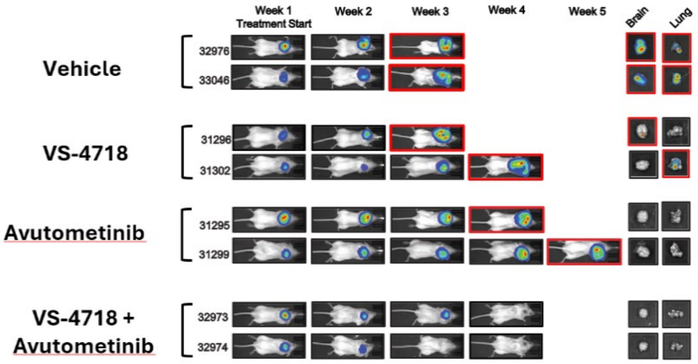

B.

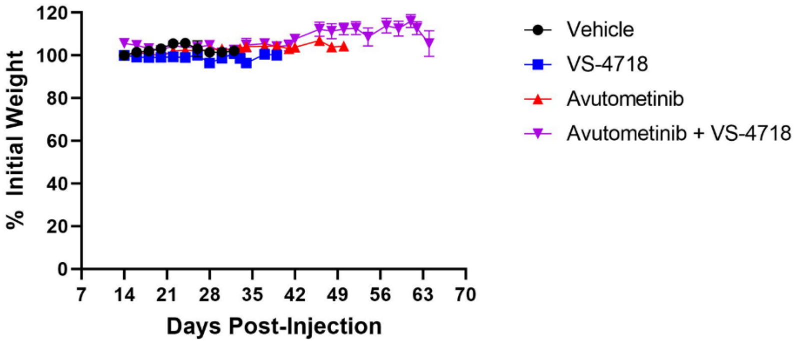

Supplemental Figure 3 title:

Combined FAK/RAF/MEK inhibition diminishes or eliminates YUMM3.2;Pten-/-;AKT1^{E17K} bioluminescence signal in the lungs and brains of mice, Related to Figure 4.

Supplemental Figure 3 legend:

A, Bioluminescence imaging (BLI) of representative 7-9 week old C57BL/6 glowing head mice subcutaneously injected with luciferase-expressing YUMM3.2;Pten-/-;AKT1^{E17K} cells. Mice were treated with vehicle, VS-4718, avutometinib, or VS-4718 + avutometinib for 28 days. BLI on the lungs and brains of mice was performed *ex vivo*, following the injection of luciferin. Red outline denotes endpoint BLI. **B,** Mean weight of mice +/-SEM by cohort over time, post-injection; vehicle (black) n=8, VS-4718 (blue) n=13, avutometinib (red) n=9, VS-4718 + avutometinib (purple) n=6.

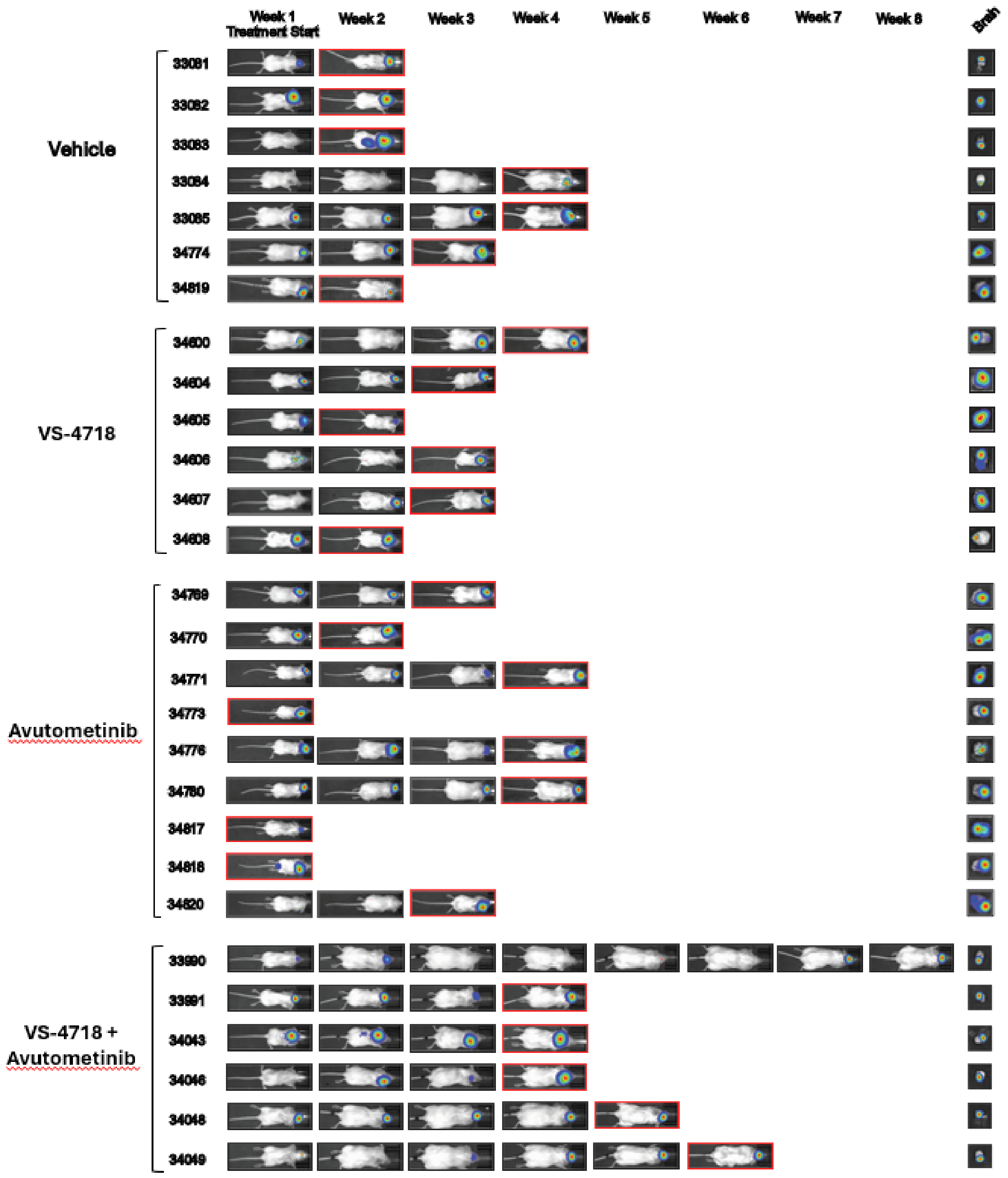

Supplemental Figure 4 title:

Combined FAK/RAF/MEK inhibition diminishes YUMM3.2;Pten-/-;AKT1^{E17K} bioluminescence signal in existing brain metastases, Related to Figure 5.

Supplemental Figure 4 legend:

Bioluminescence imaging (BLI) of newborn C57BL/6 glowing head mice intracranially injected with luciferase-expressing YUMM3.2;Pten-/-;AKT1^{E17K} cells. Mice were treated with vehicle, VS-4718, avutometinib, or VS-4718 + avutometinib for 28 days upon weaning. BLI on the brains of mice was performed *ex vivo*, following the injection of luciferin. Red outline denotes endpoint BLI.
